# Effects of a 7-Day Pornography Abstinence Period on Withdrawal-Related Symptoms in Regular Pornography Users: A Randomized Controlled Study

**DOI:** 10.1007/s10508-022-02519-w

**Published:** 2023-01-18

**Authors:** David P. Fernandez, Daria J. Kuss, Lucy V. Justice, Elaine F. Fernandez, Mark D. Griffiths

**Affiliations:** 1 Psychology Department, Nottingham Trent University, 50 Shakespeare Street, Nottingham, NG1 4FQ UK; 2 Psychology Department, HELP University, Shah Alam, Malaysia

**Keywords:** Pornography, Abstinence, Addiction, Withdrawal, Craving, Affect

## Abstract

Little is known about whether withdrawal-like symptoms manifest when regular pornography users attempt to abstain from pornography. The present study used a randomized controlled design to examine whether (1) negative abstinence effects that may be potentially reflective of withdrawal-related symptoms manifest when a non-clinical sample of regular pornography users attempt to abstain from pornography for a 7-day period and (2) these negative abstinence effects would only manifest (or manifest more strongly) for those with higher levels of problematic pornography use (PPU). A total of 176 undergraduate students (64.2% female) who were regular pornography users (defined as having used pornography ≥ three times a week in the past 4 weeks) were randomly assigned to an abstinence group (instructed to attempt abstinence from pornography for 7 days, *n* = 86) or a control group (free to watch pornography as usual, *n* = 90). Participants completed measures of craving, positive and negative affect, and withdrawal symptoms at baseline and each night of the 7-day period. Contrary to the confirmatory hypotheses, there were no significant main effects of group (abstinence vs. control) or group × PPU interaction effects on any of the outcome measures, controlling for baseline scores. These findings indicate that no evidence of withdrawal-related symptoms was found for abstaining participants, and this was not dependent on level of PPU. However, exploratory analyses showed a significant three-way interaction (group × PPU × past 4-week frequency of pornography use [FPU]) on craving, where an abstinence effect on craving was found at high levels of PPU only once past 4-week FPU reached the threshold of daily use. While these exploratory findings should be interpreted with caution, they suggest that abstinence effects could potentially manifest when there is a combination of high PPU and high FPU—a hypothesis that warrants investigation in future prospective abstinence studies.

**Supplementary Information:**

The online version contains supplementary material available at 10.1007/s10508-022-02519-w.

## Introduction

Pornography use is a common activity in the developed world, with nationally representative studies showing that 76% of men and 41% of women in Australia reported using pornography within the past year (Rissel et al., 2017), and that 47% of men and 16% of women in the USA reported using pornography at a monthly or greater frequency (Grubbs et al., 2019a).

### Problematic Pornography Use

Given the increasing prevalence of pornography use, the potential negative psychological effects of pornography use (particularly in relation to its problematic or addiction potential) have been the subject of growing empirical attention in recent years (de Alarcón et al., 2019; Grubbs & Kraus, 2021; Grubbs et al., 2020). Research to date has generally indicated that while the majority of individuals who use pornography report doing so in a non-problematic way (Bőthe et al., 2020b), a subset of users report addiction-like symptoms such as impaired control over their pornography use (Bőthe et al., 2018; Kor et al., 2014) and self-identify as being ‘addicted’ to pornography (Grubbs et al., 2018).

Despite these self-reports, researchers still disagree about whether habitual pornography users can develop genuine addictions to pornography and manifest addiction-related symptomatology akin to substance addictions. Some have argued that pornography is inherently addictive due to it being a particularly novel and rewarding stimulus (Hilton Jr., 2013) and that symptoms of dysregulated pornography use fit within an addiction framework, sharing similar neurobiological mechanisms with substance addictions and other behavioral addictions (Gola et al., 2017; Love et al., 2015), while others hold the view that pornography addiction is not a valid clinical entity and can instead be explained by non-pathological learning (Ley et al., 2014). One model (Grubbs et al., 2019b) has posited that while some users may experience genuine dysregulation in their pornography use, other users may inaccurately pathologize their use even in the absence of genuine dysregulation due to moral incongruence (i.e., using pornography while morally disapproving of it)—suggesting that self-diagnoses of “pornography addiction” should be regarded with caution.

The lack of consensus concerning how best to conceptualize dysregulated use of pornography has also been in large part due to ongoing debates in the field about how to conceptualize out-of-control sexual behaviors more broadly (Fernandez & Griffiths, 2021). There have been numerous theoretical conceptualizations of out-of-control sexual behaviors over the years, including sexual addiction (Carnes, 1983), sexual impulsivity (Barth & Kinder, 1987), compulsive sexual behavior (Coleman, 1991), and hypersexual disorder (Kafka, 2010). As a result of the varying theoretical frameworks, the term problematic pornography use (PPU) has typically been used in the literature likely because it is the most theoretically neutral term that encompasses any conceptualization of the phenomenon and includes problematic use at the subclinical end of the spectrum (Fernandez & Griffiths, 2021).

Most recently (in 2019), the World Health Organization (WHO) included the diagnosis of compulsive sexual behavior disorder (CSBD) as an impulse control disorder in the eleventh revision of the *International Classification of Diseases* (ICD-11; WHO, 2019), under which PPU may be subsumed. According to the ICD-11, CSBD is characterized by “a persistent pattern of failure to control intense, repetitive sexual impulses or urges, resulting in repetitive sexual behavior… over an extended period (e.g., six months or more) and causes marked distress or significant impairment in personal, family, social, educational, occupational or other important areas of functioning” (World Health Organization, 2019, p. 1). The ICD-11 also states that distress entirely related to moral judgments and disapproval about sexual impulses, urges or behaviors is not sufficient to meet the distress criterion. Overall, a conservative approach was taken for the ICD-11 in classifying CSBD as an impulse control disorder instead of an addictive disorder because there is (to date) insufficient evidence to determine whether the processes involved in the development and maintenance of the disorder are equivalent to other recognized forms of addiction (Kraus et al., 2018).

While the current ICD-11 CSBD diagnosis is a good starting point for unified assessment of the disorder, more research on its precise phenomenology and neurobiological underpinnings is needed to determine whether it should be re-classified as an addictive disorder in future iterations of diagnostic manuals (Griffiths, 2022; Kraus et al., 2016, 2018; Sassover & Weinstein, 2022). Determining whether CSBD (and by extension, PPU) is best conceptualized as an addictive disorder is important to ensure that appropriate pharmacological and psychological treatments are delivered depending on the specific neurobiological mechanisms and clinical features involved (Bőthe et al., 2022; Briken & Turner, 2022; Kingston, 2015; Kor et al., 2013; Lew-Starowicz & Coleman, 2022; Potenza et al., 2017). While debated (Castro-Calvo et al., 2022; Rumpf & Montag, 2022), some (Sassover & Weinstein, 2022) have proposed that to properly assess the phenomenology of CSBD, a comprehensive examination of all six components of Griffiths’ (2005) components model of addiction (i.e., salience, mood modification, conflict, withdrawal, tolerance and relapse) is needed. The present study focuses on the potential manifestation of the withdrawal component specifically in relation to PPU.

### The Assessment of Withdrawal in Relation to Problematic Pornography Use

Withdrawal can be defined as the unpleasant affective states and/or physical reactions that occur when a substance or behavior is abruptly ceased or reduced (Griffiths, 2005). Withdrawal syndromes for most addictive substances are well-established, common symptoms of which include symptoms related to emotional distress such as depressed mood, anxiety or insomnia (Shmulewitz et al., 2015), while similar withdrawal-like symptoms such as depressed mood, irritability and/or anger, and anxiety have been retrospectively self-reported in clinical samples across problematic behaviors such as gambling (e.g., Blaszczynski et al., 2008) and sexual behaviors (e.g., Wines, 1997).

In the present study, it is posited that investigating the potential manifestation of withdrawal-related symptoms in relation to PPU is important for a few reasons. First, some limited empirical research has indicated that self-perceptions of withdrawal-like symptoms are not uncommon among individuals who have attempted to abstain from pornography. A cross-sectional survey of Polish students who were current pornography users (*N* = 4260) found that among those who had at least one past pornography abstinence attempt (*n* = 2169), 72.2% recalled experiencing at least one withdrawal-like symptom upon cessation (Dwulit & Rzymski, 2019). The most commonly endorsed symptoms were erotic dreams (53.5%), irritability (26.4%) and attention disturbance (26.0%). Moreover, a recent qualitative study that analyzed abstinence journals of male members of an online pornography abstinence forum found that some members reported heightened negative affective states during abstinence, which some interpreted to possibly be withdrawal symptoms (Fernandez et al., 2021). The most commonly self-perceived withdrawal-like symptoms included depression, mood swings, anxiety, ‘brain fog,’ fatigue, headache, insomnia, restlessness, loneliness, frustration, irritability, stress and decreased motivation. These reports suggest that the possible existence of a pornography withdrawal syndrome warrants further investigation.

Second, the presence or absence of withdrawal-related symptoms in relation to PPU will inform appropriate classification of the disorder. It is worth noting that withdrawal is not considered a main component of addiction in all definitions of addiction (see, for example, Sussman & Sussman, 2011) and some scholars in the field have argued against including withdrawal in the conceptualization of behavioral addictions (e.g., Starcevic, 2016).^1^ There has also been a lack of expert consensus about the clinical relevance of withdrawal for specific behavioral addictions such as gaming disorder (Castro-Calvo et al., 2021), and withdrawal has notably not been included as a diagnostic criterion for either gaming disorder or CSBD in the ICD-11. Nonetheless, withdrawal remains an important part of key existing nosological and theoretical conceptualizations of behavioral addictions in the field. For example, withdrawal is listed as a diagnostic criterion in the *Diagnostic and Statistical Manual of Mental Disorders* (DSM-5), for the first officially recognized behavioral addiction, gambling disorder (American Psychiatric Association, 2013). Furthermore, in Griffiths’ (2005) aforementioned components model of addiction, withdrawal is one of six components that need to be present before a behavior can be diagnosed as an addiction. In sum, while withdrawal may not be necessary component of addiction in all models, the presence of withdrawal symptoms would provide additional support for potentially characterizing PPU as an addictive disorder depending on how addiction is operationalized.

Third, withdrawal is already an important component of existing theoretical conceptualizations of PPU within the empirical literature. A recent systematic review of PPU psychometric instruments found that items assessing withdrawal were included in nine of 22 instruments reviewed (Fernandez & Griffiths, 2021). One of these instruments, the Problematic Pornography Consumption Scale (PPCS; Bőthe et al., 2018, 2021b), was constructed based on Griffiths’ (2005) framework and has become a widely used measure in PPU research. Recent studies on the PPCS using network analysis have found withdrawal to be one of the central symptoms of PPU in an online sample of Hungarian men (Bőthe et al., 2020a) and the most crucial symptom of PPU among three separate samples (i.e., two community samples of Hungarian and Chinese men, and a subclinical sample of Chinese men—Chen et al., 2021). This suggests that withdrawal is a key component in the theoretical conceptualization of PPU and merits greater research attention.

Finally, it is worth acknowledging that negative psychological effects of abstinence may not necessarily be interpreted within an addiction framework as withdrawal symptoms. For example, heightened negative affect during abstinence could instead reflect normal, non-pathological reactions to sexual deprivation (Castro-Calvo et al., 2022) or a disruption of routine particularly if abstinence is involuntary or not intrinsically motivated (King et al., 2016; Szabo, 1995). Alternatively, anxiety-related effects during abstinence could be interpreted within an obsessive–compulsive framework as a consequence of being prevented from engaging in the compulsive behavior to relieve anxiety-evoking obsessive thoughts (Coleman, 1990). However, irrespective of the specific theoretical interpretation of negative abstinence effects, understanding the types of adverse reactions typically experienced by pornography users during abstinence would still be useful from a clinical standpoint as these symptoms can then become potential treatment targets in PPU interventions (Bőthe et al., 2020a).

### Methodological Limitations of Problematic Pornography Use Withdrawal Studies

To date, studies investigating withdrawal in PPU have methodological limitations that make them less than ideal for determining whether actual withdrawal symptoms manifest during periods of abstinence. Retrospective reports of negative abstinence effects during prior abstinence attempts as reported in cross-sectional surveys (e.g., Dwulit & Rzymski, 2019) are subject to recall bias (Hughes, 2007b). PPU self-report instruments that have included withdrawal items (e.g., “I became agitated when I was unable to watch porn” in the PPCS; Bőthe et al., 2018) are inherently limited if respondents typically have unrestricted access to pornography and/or never try to abstain from pornography because withdrawal symptoms, by definition, only arise under abstinence conditions (Fernandez et al., 2020; Kaptsis et al., 2016b).

Therefore, in order to properly investigate the potential manifestation of withdrawal, prospective studies of abstinence effects, preferably using intensive longitudinal methods such as ecological momentary assessment or daily diaries, are needed (Fernandez et al., 2020). Apart from observing naturally occurring abstinence situations (e.g., intrinsic quit attempts), abstinence from pornography can be experimentally manipulated to examine its effects. To date, three studies have experimentally manipulated pornography abstinence, but these have focused on its effects on relationship commitment (Lambert et al., 2012), delay discounting (Negash et al., 2016) and perceived compulsivity (Fernandez et al., 2017) rather than withdrawal-related symptoms.

### The Present Study

To address the aforementioned gaps in the literature, the present study used a randomized controlled trial (RCT) design to examine effects of a 7-day experimentally manipulated pornography abstinence period on withdrawal-related symptoms among regular pornography users. A 7-day abstinence period was chosen on the basis that the onset and peak of most substance withdrawal syndromes typically occur within the first 7 days of abstinence (Budney et al., 2003; Hughes et al., 1994; McGregor et al., 2005; Shmulewitz et al., 2015). Therefore, it can be reasonably extrapolated that withdrawal symptoms of potential behavioral addictions (if any exist) are also likely to begin manifesting within the first 7 days of abstinence. Participants were randomly assigned to one of two conditions: (1) an abstinence group, where they were given instructions to try their best to abstain from pornography for 7 days, or (2) a control group, where they were told that they are free to use pornography as usual. Outcome measures were assessed at baseline the night before the start of the experimental period (retrospectively about the past 7 days) and each night of the experimental period using end-of-day online surveys.

Three potential manifestations of withdrawal-related symptoms were examined in the present study. First, craving for pornography was examined in light of findings from a recent systematic review that craving was the most common abstinence effect across multiple behaviors including gambling, gaming, mobile phone use and social media use (Fernandez et al., 2020). Therefore, it would be logical to examine whether craving is also an abstinence effect for pornography use. Second, positive and negative affect was examined because affective disturbances are common self-perceived withdrawal-like symptoms reported by pornography users (Dwulit & Rzymski, 2019; Fernandez et al., 2021). Increased negative affect and/or decreased positive affect may be a possible manifestation of withdrawal, in line with previous prospective studies of abstinence from substances such as nicotine (e.g., Hughes, 2007a; Klemperer et al., 2021) and behaviors such as exercise (Fernandez et al., 2020). Third, *withdrawal symptoms* as assessed by an adaptation of the Wisconsin Smoking Withdrawal Scale (Welsch et al., 1999) for pornography use were examined because retrospective survey and qualitative research has shown that the more commonly self-reported withdrawal-like symptoms by pornography users (i.e., depressed mood, irritability/frustration, anxiety and difficulty in concentrating—Dwulit & Rzymski, 2019; Fernandez et al., 2021) overlap to a considerable extent with symptoms of cigarette-smoking withdrawal (Hughes, 2007a).

A non-clinical sample of regular pornography users was used instead of a clinical sample (e.g., treatment-seeking individuals) because withdrawal-like symptoms have been shown to manifest during abstinence from other behaviors (i.e., exercise, social media use, mobile phone use and gaming) even for regular users who have no apparent indication of problematic use (Fernandez et al., 2020). Several authors (e.g., Fernandez et al., 2020; Kaptsis et al., 2016a) have also recommended against using clinical samples in abstinence studies until any possible negative abstinence effects are first understood in less complex populations. Nonetheless, within a non-clinical sample of regular users, it would be expected that those with higher self-reported levels of PPU would experience more pronounced negative abstinence effects, if any.

Taken together, the present study sought to answer the following two broad research questions: (1) Do negative abstinence effects (potentially indicative of withdrawal-related symptoms) manifest when regular pornography users try to abstain from pornography for a 7-day period? and (2) Do these negative abstinence effects only manifest (or manifest more strongly) for those with higher levels of PPU? If a pornography withdrawal syndrome were assumed to exist, the following hypotheses would be expected to be supported:

#### H_1_

There are significant main effects of group (abstinence vs. control) on craving, positive affect, negative affect and withdrawal symptoms, controlling for baseline scores. More specifically, compared to the control group, the abstinence group are expected to score significantly higher on craving, negative affect and withdrawal symptoms, and significantly lower on positive affect.

#### H_2_

There is a significant group × PPU interaction on craving, positive affect, negative affect and withdrawal symptoms, controlling for baseline scores. More specifically, it is predicted that abstinence effects either (a) only manifest for those with higher PPU and not for those with lower PPU, or (b) manifest more strongly for those with higher PPU compared to those with lower PPU.

## Method

### Participants

Participants were psychology undergraduate students at a university in Malaysia. An a priori power analysis using the *pwr.f2.test* function in R determined that to detect an effect size of *f*^2^ = 0.06 (Cohen’s *d* = 0.5), assuming alpha = 0.05, power = 0.8 and numerator degrees of freedom = 2, a sample size of 164 participants was required. Cohen’s *d* = 0.5 was set as the smallest effect size of interest (Lakens, 2022) for the present study because it has been specified in the literature to be the smallest difference individuals are able to detect in health-related quality of life outcomes (Norman et al., 2003).

Eligibility criteria for participation included: (1) being at least 18 years old and (2) being a regular pornography user, operationally defined here as having watched pornography at least three times a week in the 4 weeks leading up to the study start date. While this definition of “regular” pornography use is arbitrary, it was reasoned that this minimum frequency was needed for a 7-day abstinence period to represent a significant enough deprivation of participants’ weekly pornography use habits to be able to induce potential withdrawal symptoms. A custom definition of pornography adapted and modified from definitions found in the past literature (Grubbs et al., 2019a; McKee et al., 2020) was provided for participants as follows: “any sexually explicit films, video clips or pictures which intend to sexually arouse the viewer; this may be seen on the internet, in a magazine, in a book, or on television.” While males report higher rates of pornography use (e.g., Hald, 2006; Kvalem et al., 2014; Regnerus et al., 2016) and PPU (e.g., Grubbs et al., 2019a; Kor et al., 2014) than females, there is no evidence to suggest that withdrawal symptoms, if any exist, would manifest only for male pornography users. Previous cross-sectional research found that both males and females recalled experiencing withdrawal-like symptoms during a past cessation attempt (Dwulit & Rzymski, 2019). Therefore, there was no gender restriction for participating in the present study.

**Fig. 1:**
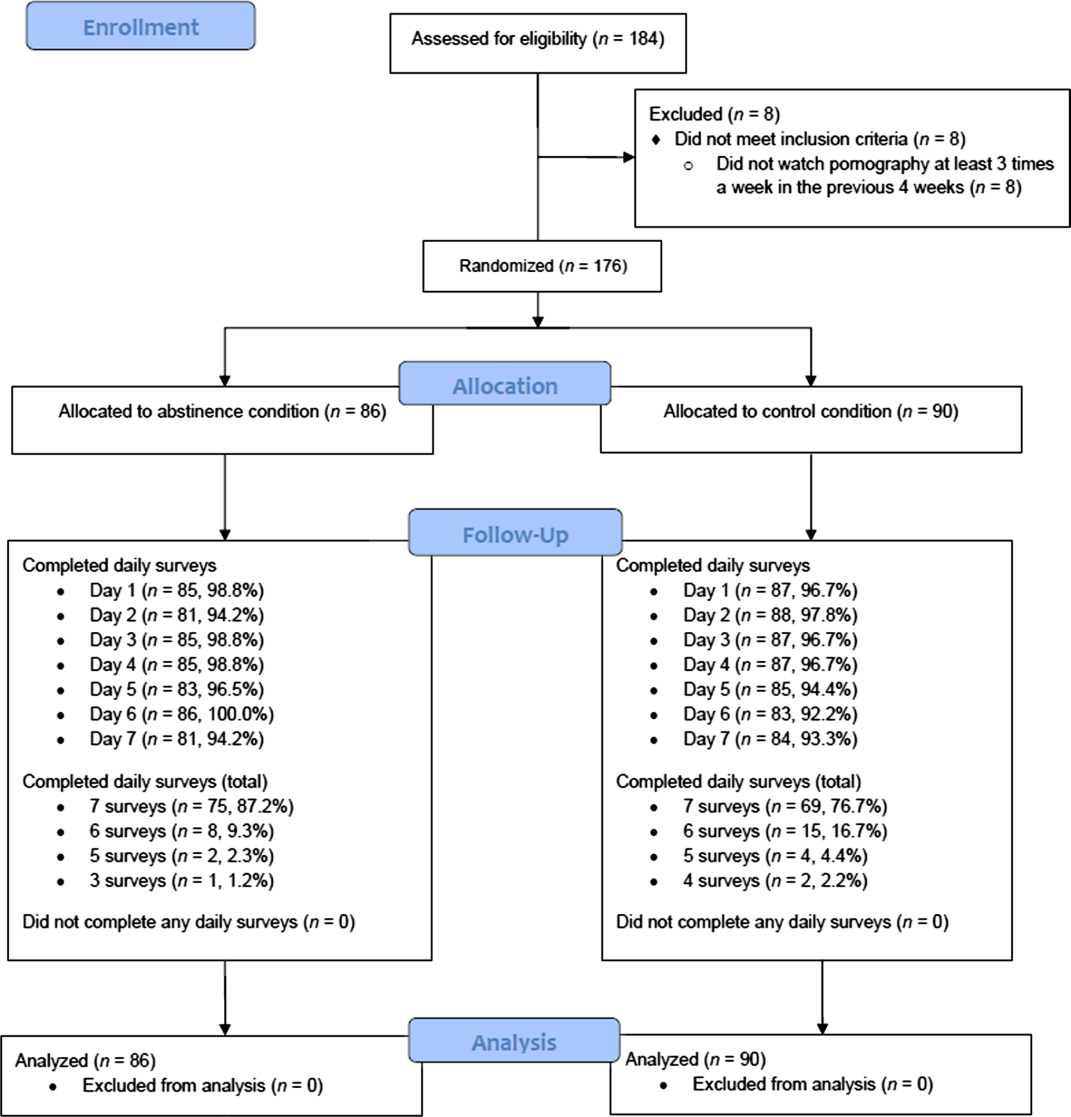
CONSORT flow diagram

A total of 184 students signed up for the study and were assessed for eligibility through their responses to questions on the baseline survey. Eight participants were excluded at this stage because they did not meet the requirement of being a regular pornography user. A total of 176 participants were randomized, resulting in 86 participants allocated to the abstinence group and 90 participants allocated to the control group. No participants failed to complete any daily survey post-randomization (144 completed all seven surveys, 23 completed six surveys, six completed five surveys, two completed four surveys, and one completed three surveys). Daily survey completion rate across the 7 days ranged from 92.2% to 100.0%. An intention-to-treat strategy (Gupta, 2011) to the analysis was applied. Therefore, all randomized participants were retained for data analysis. The final sample comprised 176 participants (64.2% female, 34.7% male, 1.1% agender, *M*_age_ = 21.38 years, SD = 1.50). Figure 1 depicts a Consolidated Standards of Reporting Trials (CONSORT) flow chart detailing the flow of participants through each stage of the study.

Baseline characteristics of the sample are presented in Table S1 in Supplementary Material. At baseline, 48.9% of participants reported desire to reduce but not completely quit their pornography use, 40.9% of participants reported no desire to reduce nor quit their pornography use, and 10.2% reported desire to completely quit their pornography use. In line with CONSORT best practice recommendations for RCTs (Altman et al., 2001), significance tests of baseline differences between the groups were not conducted as due to the randomization procedure, any potential observed baseline differences would be attributable to chance (de Boer et al., 2015; Harvey, 2018).

It is important to highlight that Malaysia, where the present study was conducted, is a sexually conservative country with restrictive laws regulating pornography use (e.g., access to pornographic websites is blocked by the government) and with religious and cultural values that may influence perception of pornography use as immoral or a sin (Goh et al., 2019; Tan et al., 2022). Despite this conservatism, empirical research has shown that pornography use is not uncommon among university students in Malaysia (Ali et al., 2021). Of note, it appears that most participants in the present sample had relatively liberal attitudes toward pornography use as indicated by the low rate of agreement in the baseline survey with the statement “I believe that pornography use is morally wrong” (i.e., 67.6% disagreed at least to some extent, 18.2% neither agreed nor disagreed, and only 14.2% agreed at least to some extent). Nonetheless, due to the conservative sociocultural context, the possibility of additional self-selection bias and social desirability bias (potentially leading to underreporting of pornography use and sexual behaviors) among participants needs to be acknowledged.

### Procedure

**Fig. 2:**
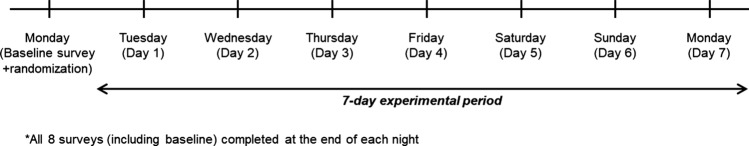
Timeline of the 8-day study period

The study was advertised to prospective participants as “The pornography abstinence experiment: An 8-day daily diary study of regular pornography users.” Participants were informed that the purpose of the study would be to track the daily experiences of regular pornography users who will be randomly assigned either to an “abstinence” group or a “no abstinence” group.^2^ Three separate 8-day sessions of the study were conducted over the course of 2 months in 2021 (one in February and two in March). All three sessions began on a Monday and ended on the following Monday (see Fig. 2). Participants were required to complete a daily survey each night during the 8-day period (including the baseline survey on the first Monday). At 8.00 pm each night of the 8-day period, online *Qualtrics* survey links were emailed to participants. Participants were asked to complete the surveys at the end of their day, as close as possible before they went to bed. The survey links expired at 5.00 am the next morning to ensure that participants did not complete the surveys after waking.

After completing the baseline survey, participants were randomly allocated to the abstinence and control groups by the *Qualtrics* randomizer function, which was set to assign a roughly equal number of participants to each condition.^3^ Participants in the abstinence group received instructions to try their best to abstain from pornography for the next 7 days, starting from 5.00 am the next morning (Tuesday) to 5.00 am the following Tuesday morning (exactly 7 days). Participants were told that if they did watch pornography during this period, to report it honestly during the daily surveys, and that it would not have an effect on any compensation they were entitled to receive. They were also told that they were allowed to masturbate without pornography or engage in any other non-pornography-related sexual activity during this period. The control group were told that they were free to watch pornography as usual for the next 7 days (see Appendix A in Supplementary Material for full instructions provided to the abstinence and control groups).

As compensation, participants received course credit if they completed at least six of the seven surveys during the experimental period, and in addition received a gift voucher worth MYR 30.00 (approximately 7.00 USD) if they completed all seven surveys. Informed consent was obtained from all participants, and all study procedures were approved by the research team’s university ethics committees.

### Measures

All measures used in the present study are provided in their full form in Appendix B in Supplementary Material and are summarized below.

#### Daily Measures

***Daily frequency and/or duration of sexual behavior*** During each daily survey, five items asked about the frequency and/or duration of sexual behaviors participants engaged in during two separate time windows: (1) after completing last night’s survey and before going to bed and (2) since waking up that day. This was to ensure that any sexual behavior engaged in after each nightly survey was also accounted for. For all five items, daily totals were computed by summing the frequency/duration reported about both time frames that made up one full day: (1) since waking up that day and (2) after completing that night’s survey and before going to bed (reported on the following day’s survey). These five items are summarized as follows:

*Daily frequency of pornography use (FPU)* Two free response items asked participants to report how many times they had (1) watched pornography without masturbating and (2) watched pornography while masturbating, respectively. Daily FPU was computed by summing the frequencies reported on both items.

*Daily duration of pornography use* A third free response item asked participants to report (in minutes) how much time in total they had spent watching pornography.

*Daily frequency of masturbation without pornography* A fourth free response item asked participants to report how many times they had masturbated without watching pornography.

*Daily frequency of alternative sexual activity* A fifth free response item asked participants to report how many times they had engaged in any sexual activity other than pornography use or masturbation (e.g., oral sex, intercourse).

***Daily abstinence effort*** Daily abstinence effort was assessed using a single item adapted from a previous pornography abstinence study (Fernandez et al., 2017) that asked participants to rate the extent to which they were trying their best to abstain from pornography use since waking up that day, on a 10-point scale (0 = “I did not try at all” to 10 = “I tried my best”).

***Daily craving*** Daily craving was assessed using a modified four-item version of the five-item Penn Alcohol Craving Scale (PACS; Flannery et al., 1999), adapted for pornography use.^4^ Four PACS items were modified to refer to pornography use instead of alcohol and asked participants to rate their craving for pornography since waking up that day in terms of frequency of thoughts about pornography, intensity of craving at its strongest point, difficulty in resisting pornography and average craving since waking. A fifth item from the original PACS assessing the duration of thoughts of alcohol was excluded on the basis that it would be challenging for participants to distinguish craving frequency from craving duration when reporting on a 24-h period (Hallgren et al., 2018). Responses were indicated on a seven-point scale, with response options differing according to each item. The scale demonstrated excellent internal consistency across all time points (α = 0.90–0.97).

***Daily positive and negative affect*** Daily positive and negative affect was assessed using the 10-item International Positive and Negative Affect Schedule-Short Form (I-PANAS-SF; Thompson, 2007). The I-PANAS-SF comprises two five-item subscales, positive affect and negative affect, which are each composed of a list of adjectives describing positive (e.g., “inspired”) and negative (e.g., “upset”) affective states, respectively. Participants rated the extent to which they felt these affective states since waking up that day on a five-point scale (1 = “very slightly or not at all” to 5 = “extremely”). Internal consistency was very good across all time points for both positive affect (α = 0.80–0.88) and negative affect (α = 0.80–0.86) subscales.

***Daily withdrawal symptoms*** Daily withdrawal symptoms were assessed using a modified 14-item version of the 28-item Wisconsin Smoking Withdrawal Scale (WSWS; Welsch et al., 1999), adapted for pornography use. The 28 original WSWS items are divided into seven subscales (anger, anxiety, concentration, craving, hunger, sadness and sleep). Only items from four of the subscales (anger, anxiety, concentration and sadness) were retained as these mapped onto the more commonly self-reported categories of withdrawal-like symptoms in previous pornography studies (i.e., irritability/frustration, anxiety, difficulty in concentrating and depressed mood; Dwulit & Rzymski, 2019; Fernandez et al., 2021). The modified scale comprised 14 items which were composed of statements describing specific symptoms (e.g., “It has been difficult to think clearly”). Participants rated their agreement with these statements on a five-point scale (0 = “strongly disagree” to 4 = “strongly agree”) based on how they felt in general since waking up that day. A composite score was computed based on the mean score of all the items. The scale demonstrated excellent internal consistency across all time points (α = 0.91–0.93).

#### Baseline Measures

*Demographic information* Participants reported demographic information at baseline, including gender, age, sexual orientation, relationship status and nationality.

*Past 4-week frequency of pornography use (FPU)* One item assessed participants’ FPU over the previous 4 weeks on a seven-point scale (0 = “less than 3 times a week on average” to 6 = “more than once a day on average”).

*Past 4-week average duration of pornography use per session* One item assessed participants’ average duration of pornography use per session over the previous 4 weeks on a 10-point scale (1 = “5 min or less” to 10 = “3 h or more”).

*Past 4-week frequency of masturbation without pornography* One item assessed participants’ frequency of masturbation without pornography over the past 4 weeks on a six-point scale (1 = “never” to 6 = “about 7 or more times a week on average”).

*Past 4-week percentage of pornography use accompanied by masturbation* One item asked participants to estimate what percentage (from 0 to 100) of all their pornography watching sessions in the past 4 weeks was accompanied by masturbation.

*Past 7-day frequency of pornography use (FPU)* Two free response items asked how many times over the past 7 days participants had (1) watched pornography without masturbating and (2) watched pornography while masturbating, respectively. Past 7-day FPU was computed by summing the frequencies reported on both items.

*Past 7-day duration of pornography use* Past 7-day duration of pornography use was assessed by one free response item that asked participants to report how much time in total (in minutes) they watched pornography over the past 7 days.

*Baseline abstinence effort (past 7 days)* Baseline abstinence effort was assessed using the same single item used to assess daily abstinence effort, but asked participants to rate the extent to which they were trying their best to abstain from pornography use over the past 7 days.

*Baseline craving (past 7 days)* Baseline craving was assessed using the same four PACS items used to assess daily craving, but asked participants to rate their craving for pornography during the previous 7 days. The scale demonstrated very good internal consistency (α = 0.81).

*Baseline positive and negative affect (past 7 days)* Baseline positive and negative affect was assessed using the 10-item I-PANAS-SF, but asked participants to rate their positive and negative affect during the past 7 days. Internal consistency was fair for both positive affect (α = 0.77) and negative affect (α = 0.77) subscales.

*Baseline withdrawal symptoms (past 7 days)* Baseline withdrawal symptoms were assessed using the same modified 14-item WSWS used to assess daily withdrawal symptoms, but asked participants to rate their withdrawal symptoms during the past 7 days. The scale demonstrated very good internal consistency (α = 0.89).

*Problematic pornography use (PPU)* PPU was assessed using the 18-item Problematic Pornography Consumption Scale (PPCS; Bőthe et al., 2018) (α = 0.92). PPCS assesses past 6-month PPU covering six factors: salience, tolerance, mood modification, withdrawal, relapse and conflict (Griffiths, 2005) on a seven-point scale (1 = “never” to 7 = “all the time”). Higher scores indicate greater problematic pornography consumption.

*Intrinsic desire to quit or reduce pornography use* One item assessed whether or not participants intrinsically desired to quit or reduce their pornography use at the time of the baseline survey. Participants indicated their agreement with one of the following three response options: “I want to reduce my pornography use, but I don’t want to completely quit/stop using pornography,” “I want to completely quit/stop using pornography” or “I have no desire to reduce or completely quit/stop using pornography.”

*Moral disapproval of pornography* Moral disapproval of pornography was assessed using the single item “I believe that pornography use is morally wrong” used in previous studies (Grubbs et al., 2019a) rated on a seven-point scale (1 = “strongly disagree” to 7 = “strongly agree”).

#### Validity of Outcome Measures

Since the outcome measures used in the present study (four-item PACS adapted for pornography use, I-PANAS-SF and 14-item WSWS adapted for pornography use) were not developed for the purpose of assessing pornography withdrawal-related symptoms, their structural and construct validity were briefly examined. First, confirmatory factor analyses were conducted on each baseline outcome measure to examine their factor structure in the present sample. Second, associations of the outcome measures with each other and with pornography use variables at baseline were examined to assess their convergent and discriminant validity. Results of these analyses are reported in detail in Appendix C and Tables S2-S5 in Supplementary Material. Overall, the outcome measures appeared to demonstrate adequate structural and construct validity for the purposes of the present study.

#### Attention Check Measures

To screen for inattentive responding and to encourage attentive responding, attention check items (i.e., “Please select [specific answer] to this question”; Shamon & Berning, 2020) were embedded in the surveys (one in the baseline survey and two in each daily survey). If participants selected the wrong answer on these items, they were alerted with a pop-up message encouraging them to remain attentive throughout the rest of the survey.

### Data Analysis

Prior to conducting any substantive analyses, the dataset was screened for careless responding based on several criteria typically used in online survey research. First, examination of responses to the attention check items showed that all participants passed more than half of all attention checks, with 95% of the sample having an attention check pass rate of ≥ 88%. Second, an examination of response time per item indicated that there were no participants who completed any of the surveys too quickly (i.e., quicker than two seconds per item—Huang et al., 2012). Third, long-string analysis indicated that two participants appeared to engage in invariant responding on one of the scales in one of the daily surveys (i.e., providing a string of consistent responses on more than half of the scale—Curran, 2016). However, these two participants had a 100% attention check pass rate across all the surveys. Therefore, when considered together, none of the participants were flagged for careless responding.

All analyses were performed within the R environment (R Core Team, 2021). Due to the nested nature of the data (daily measures [level 1] nested within participants [level 2]) multilevel modeling (MLM) was utilized to examine group differences. Multilevel models model all available data from all participants regardless of missing observations and as such are robust to missing data. MLM is consistent with the intent-to-treat approach as all participants are retained in the analysis (listwise deletion is not required).

Models with continuous data (e.g., craving) were fit using the *lmer()* function in the *lme4* package (Bates et al., 2015) with restricted maximum likelihood (REML) estimation. The *p*-values were estimated with the *lmertest* package using Satterthwaite’s degrees of freedom method (Kuznetsova et al., 2017). These models were checked to verify that they met assumptions of linearity, homoscedasticity and normality using a residuals versus fitted values plot, a scale location plot and a normal probability plot, respectively. The plots indicated that these assumptions were met for all models, except for the negative affect model, which indicated deviation from normality. Negative affect was log-transformed to reduce skewness and approximated a normal distribution after transformation. Models with count data (e.g., FPU) were fit using the *glmer()* function in the *lme4* package using a Poisson distribution, while the model with ordinal data (i.e., abstinence effort) was fit using the *clmm()* function from the *ordinal* package (Christensen, 2019).

For all models, the maximal random effects structure (Barr et al., 2013) was used, with a random intercept for participant and a random slope for time (i.e., Time | Participant), which allowed each participant to have their own intercept and slope across time (days). Where models failed to converge while using the default optimizer in *lme4*, models were refit with all available optimizers (Bates et al., 2015). If models converged and produced similar results while using the other optimizers, the non-convergence warnings were regarded as false positives and an alternative optimizer was used instead. If models still failed to converge while using the other optimizers, the random effects structure was simplified to 1 | Participant (by removing the random slope term) to facilitate model convergence.^5^

For all confirmatory and exploratory analyses,^6^ all predictors and interactions were first entered into an initial “full” model. Using a backward elimination procedure, reduced models were specified by removing nonsignificant predictors. At each step, the more parsimonious model was compared to the previous model using the *anova()* function, which computes *χ*^2^ statistics representing the differences in deviance between the two models with corresponding *p*-values based on likelihood ratio test comparisons (Bates et al., 2015). This process was repeated until the best-fitting model was identified for each separate outcome measure. The baseline score of each outcome measure,^7^ frequency of masturbation without pornography and frequency of alternative sexual activity were controlled for as covariates in all confirmatory and exploratory models. Given the relatively broad definition of regular pornography use in the present study (≥ three times a week), past 4-week FPU was also included as a covariate in all confirmatory models. Results of key best-fitting models are presented in Tables 1 and 2 in Results section, while all model fit and comparison statistics are presented in Tables S8-9, S11-12 and S15-16 in supplementary material.

**Table 1:** Multilevel model results for all outcome variables in confirmatory analyses

Outcome variable	Fixed effect	Estimate (*SE*)	*df*	*t*	*p*	95% CI	Semi-partial *R*^2^
Craving	Intercept	0.48 (0.97)	169.57	0.49	0.626	− 1.43–2.38	
	Group	− 0.34 (0.50)	165.38	− 0.68	0.498	− 1.32–0.64	0.003
	PPU	0.09 (0.02)	164.16	4.45	** < 0.001**	0.05–0.13	0.108
	Baseline craving	0.08 (0.10)	162.89	0.85	0.394	− 0.11–0.27	0.004
	Past 4-week FPU	0.28 (0.16)	171.74	1.67	0.096	− 0.05–0.60	0.016
	Frequency of masturbation without pornography	0.46 (0.30)	937.60	1.53	0.127	− 0.13–1.04	0.002
	Frequency of alternative sexual activity	− 0.33 (0.34)	1008.16	− 0.97	0.334	− 0.99–0.34	0.001
	Time	− 0.06 (0.07)	169.49	− 0.86	0.392	− 0.21–0.08	0.004
Positive affect	Intercept	4.92 (0.96)	177.57	5.10	** < 0.001**	3.03–6.81	
	Group	− 0.34 (0.40)	170.18	− 0.85	0.396	− 1.13–0.45	0.004
	Baseline positive affect	0.59 (0.05)	172.79	10.83	** < 0.001**	0.48–0.69	0.405
	Past 4-week FPU	− 0.03 (0.12)	174.38	− 0.21	0.832	− 0.27–0.22	0.000
	Frequency of masturbation without pornography	− 0.23 (0.21)	1042.37	− 1.13	0.260	− 0.64–0.17	0.001
	Frequency of alternative sexual activity	0.20 (0.23)	979.97	0.86	0.389	− 0.25–0.64	0.001
	Time	0.07 (0.05)	168.00	1.37	0.172	− 0.03–0.17	0.011
Negative affect^a^	Intercept	1.49 (0.08)	172.69	18.19	** < 0.001**	1.33–1.65	
	Group	0.00 (0.04)	165.79	0.12	0.906	− 0.07–0.08	0.000
	Baseline negative affect	0.05 (0.00)	166.29	11.56	** < 0.001**	0.04–0.06	0.445
	Past 4-week FPU	0.01 (0.01)	168.72	0.80	0.425	− 0.01–0.03	0.004
	Frequency of masturbation without pornography	− 0.00 (0.02)	1062.73	− 0.19	0.850	− 0.04–0.03	0.000
	Frequency of alternative sexual activity	0.02 (0.02)	975.31	0.94	0.347	− 0.02–0.06	0.001
	Time	− 0.01 (0.00)	167.64	− 2.68	**0.008**	− 0.02 to − 0.00	0.041
Withdrawal symptoms	Intercept	0.35 (0.15)	167.19	2.18	**0.031**	0.03–0.66	
	Group	0.10 (0.07)	164.02	1.42	0.157	− 0.04–0.24	0.012
	PPU	0.01 (0.00)	165.11	2.61	**0.010**	0.00–0.01	0.040
	Baseline withdrawal symptoms	0.58 (0.05)	169.21	11.40	** < 0.001**	0.48–0.68	0.434
	Past 4-week FPU	− 0.02 (0.02)	168.35	− 1.03	0.305	− 0.07–0.02	0.006
	Frequency of masturbation without pornography	0.02 (0.04)	1023.51	0.56	0.576	− 0.05–0.10	0.000
	Frequency of alternative sexual activity	− 0.04 (0.04)	990.12	− 0.86	0.389	− 0.12–0.05	0.001
	Time	− 0.03 (0.01)	172.70	− 3.28	**0.001**	− 0.05 to − 0.01	0.061

^a^Log-transformed. All models were random slope models with Time | Participant random effects. The best-fitting models are presented. *CI* confidence interval, *FPU* frequency of pornography use, *PPU* problematic pornography use, *SE* standard error

**Table 2:** Multilevel model results for all outcome variables in exploratory analyses with past 4-week FPU as moderator

Outcome variable	Fixed effect	Estimate (*SE*)	*df*	*t*	*p*	95% CI	Semi-partial R^2^
Craving	Intercept	5.41 (2.84)	160.01	1.90	0.059	− 0.17–10.98	
	Group	− 7.36 (3.61)	160.97	− 2.04	**0**.**043**	− 14.45 to − 0.27	0.025
	PPU	− 0.04 (0.05)	160.81	− 0.78	0.437	− 0.13–0.06	0.017
	Past 4-week FPU	− 0.93 (0.79)	158.53	− 1.17	0.244	− 2.48–0.63	0.002
	Gender	0.44 (0.57)	160.82	0.77	0.445	− 0.68–1.56	0.004
	Baseline craving	0.07 (0.09)	158.10	0.70	0.488	− 0.12–0.25	0.003
	Frequency of masturbation without pornography	0.37 (0.30)	905.33	1.26	0.209	− 0.21–0.96	0.002
	Frequency of alternative sexual activity	− 0.37 (0.34)	997.84	− 1.09	0.277	− 1.03–0.30	0.001
	Time	− 0.05 (0.07)	170.18	− 0.68	0.496	− 0.19–0.09	0.003
	Group × PPU	0.19 (0.06)	161.66	2.93	**0**.**004**	0.06–0.32	0.050
	Group × past 4-week FPU	1.22 (1.01)	160.69	1.21	0.228	− 0.76–3.19	0.009
	PPU × past 4-week FPU	0.03 (0.01)	159.08	2.46	**0**.**015**	0.01–0.05	0.013
	Group × PPU × past 4-week FPU	− 0.04 (0.02)	160.43	− 2.30	**0**.**023**	− 0.07 to − 0.01	0.032
Positive affect	Intercept	4.65 (1.15)	171.94	4.03	**< **.**0001**	2.39–6.91	
	Group	− 0.39 (0.41)	167.43	− 0.95	0.342	− 1.18–0.41	0.005
	Gender	0.13 (0.49)	167.14	0.26	0.795	− 0.86–1.08	0.000
	Baseline positive affect	0.59 (0.05)	170.30	10.84	**< 0**.**001**	0.49–0.70	0.408
	Frequency of masturbation without pornography	− 0.24 (0.21)	1032.06	− 1.17	0.244	− 0.65–0.16	0.001
	Frequency of alternative sexual activity	0.19 (0.23)	969.39	0.86	0.389	− 0.25–0.64	0.001
	Past 4-week FPU	0.01 (0.14)	169.09	0.10	0.924	− 0.27–0.29	0.000
	Time	0.07 (0.05)	167.82	1.28	0.201	− 0.04–0.17	0.010
Negative affect^a^	Intercept	1.29 (0.10)	163.05	12.50	**< 0**.**001**	1.09–1.50	
	Group	0.01 (0.04)	160.87	0.34	0.736	− 0.06–0.09	0.001
	PPU	0.00 (0.00)	161.39	2.09	**0.039**	0.00–0.00	0.026
	Past 4-week FPU	0.01 (0.01)	162.45	1.00	0.318	− 0.01–0.04	0.006
	Gender	0.10 (0.05)	161.44	2.19	**0**.**030**	0.01–0.20	0.029
	Baseline negative affect	0.05 (0.00)	162.00	10.76	**< 0**.**001**	0.04–0.06	0.417
	Frequency of masturbation without pornography	− 0.00 (0.02)	1046.72	− 0.09	**0**.**930**	− 0.04–0.04	0.000
	Frequency of alternative sexual activity	0.02 (0.02)	964.45	0.86	**0**.**392**	− 0.02–0.06	0.001
	Time	− 0.01 (0.00)	167.42	− 2.61	**0**.**010**	− 0.02 to − 0.00	0.039
Withdrawal symptoms	Intercept	0.19 (0.18)	162.50	1.04	0.301	− 0.17–0.55	
	Group	0.10 (0.07)	161.48	1.34	0.181	− 0.04–0.24	0.011
	PPU	0.01 (0.00)	162.22	2.75	**0**.**007**	0.00–0.01	0.045
	Past 4-week FPU	− 0.00 (0.03)	163.66	− 0.15	0.885	− 0.05–0.05	0.000
	Gender	0.15 (0.09)	161.93	1.75	0.082	− 0.02–0.32	0.019
	Baseline withdrawal symptoms	0.57 (0.05)	166.33	10.81	**< 0**.**001**	0.46–0.67	0.413
	Frequency of masturbation without pornography	0.02 (0.04)	1014.50	0.64	0.521	− 0.05–0.10	0.000
	Frequency of alternative sexual activity	− 0.04 (0.04)	980.29	− 0.94	0.347	− 0.12–0.04	0.001
	Time	− 0.03 (0.01)	166.64	− 3.18	**0**.**002**	− 0.05 to − 0.01	0.057

^a^Log-transformed. One participant in the abstinence group and one participant in the control group who identified as “agender” were excluded from these analyses, resulting in *N* = 174. All models are random slope models with Time | Participant random effects. The best-fitting models are presented. *CI* confidence interval, *FPU* frequency of pornography use, *PPU* problematic pornography use, *SE* standard error

Effect sizes of individual fixed effects were determined by computing the semi-partial *R*^2^ statistic (Jaeger et al., 2017) through the *r2beta* function in the *r2glmm* package (Jaeger, 2017) using the Kenward–Roger approach. The semi-partial *R*^2^ statistic represents the unique proportion of variance explained by each individual predictor in the model, above and beyond the variance explained by all other predictors in the model. Wherever a final model included a significant interaction, post hoc analyses were conducted to clarify the nature of the interaction using the *emmeans* package (Lenth, 2021). The *emmeans* function was used to compute estimated marginal means and conduct pairwise comparisons of interest, while the *emmip* function was used to create interaction plots using estimated marginal means.

## Results

### Manipulation Check Analyses

To determine whether participants in the abstinence group adhered to instructions to try their best to abstain from pornography during the experimental period, group differences in abstinence effort, FPU and duration of pornography use during the experimental period were examined (see Table S6 in Supplementary Material). The abstinence group reported significantly greater daily abstinence effort (*M* = 4.72, SD = 3.77) compared to the control group (*M* = 0.96, SD = 2.39), controlling for past 7-day abstinence effort (odds ratio = 0.01, *z* =  −  9.37, *p* < 0.001). The abstinence group also reported significantly lower daily FPU (*M* = 0.27, SD = 0.80) compared to the control group (*M* = 0.93, SD = 1.04), controlling for past 7-day FPU (incidence rate ratio [IRR] = 4.95, *z* = 9.38, *p* < 0.001). The abstinence group also reported significantly lower daily duration (in minutes) of pornography use (*M* = 4.25, SD = 15.30) compared to the control group (*M* = 16.91, SD = 23.05), controlling for past 7-day duration of pornography use (*b* = 12.70, *t*[171.76] = 6.97, *p* < 0.001). Taken together, these group differences indicate that the abstinence instructions were effective at inducing attempts to abstain from pornography in the abstinence group during the experimental period.

While the majority of participants in the abstinence group (*n* = 47/86; 54.65%) did not report any pornography use at all during the experimental period, a considerable proportion of participants (*n* = 39/86; 45.35%) reported using pornography at least once during this period. More specifically, seven participants (8.14%) reported using pornography once, eleven (12.79%) reported using pornography twice, nine reported using pornography three times (10.47%) and twelve (13.95%) reported using pornography four or more times. For these participants, pornography use during the experimental period might be indicative of two possibilities: (1) a lack of adherence to instructions to try their best to abstain from pornography and/or (2) a failure to abstain from pornography despite attempting to do so (i.e., a “lapse”). These participants were not excluded from the analyses for two reasons. First, the intention-to-treat principle states that all randomized participants should be included in analyses according to their originally assigned condition, regardless of protocol adherence in order to maintain comparability between groups obtained through randomization and minimize the risk of bias (Gupta, 2011). Second, the purpose of the abstinence instructions was to induce attempts at abstinence (which is within participants’ control) and not necessarily the achievement of successful nonuse (which may not entirely be within all participants’ control, given the difficulty that may come with trying to regulate habitual pornography use for 7 days). Lapses are common in real-world pornography abstinence attempts (Fernandez et al., 2021), and therefore, emphasizing attempted abstinence over successful nonuse may arguably have greater ecological validity. Furthermore, nicotine abstinence research has demonstrated that excluding participants who relapse from analyses may be counterproductive because these participants are most likely to experience the greatest amount of withdrawal in the first place (see Hughes, 2007b; Piasecki, et al., 2003a, 2003b; Shiffman et al., 2004).

### Confirmatory Analyses

Means and standard deviations for all study variables are presented in Table S7 in Supplementary Material.

Results of the MLM analyses (see Table 1) showed no statistically significant main effects of group on craving (*b* = − 0.34, *t*[165.38] = − 0.68, *p* = 0.498, semi-partial *R*^2^ = 0.003), positive affect (*b* = − 0.34, *t*[170.18] = − 0.85, *p* = 0.396, semi-partial *R*^2^ = 0.004), negative affect (*b* = 0.00, *t*[165.79] = 0.12, *p* = 0.906, semi-partial *R*^2^ = 0.000) or withdrawal symptoms (*b* = 0.10, *t*[164.02] = 1.42, *p* = 0.157, semi-partial *R*^2^ = 0.012), controlling for baseline scores, past 4-week FPU, frequency of masturbation without pornography and frequency of alternative sexual activity. This means that contrary to H_1_, there were no significant differences between the abstinence and control groups on any of the outcome measures. The addition of a group × PPU interaction term did not improve model fit (Tables S8-S9 in Supplementary Material) for the craving model (*χ*^2^ = 1.35, *p* = 0.245), positive affect model (*χ*^2^ = 1.57, *p* = 0.210), negative affect model (*χ*^2^ = 0.59, *p* = 0.443) or withdrawal symptoms model (*χ*^2^ = 1.50, *p* = 0.220). This means that contrary to H_2_, there was no significant interaction between group and PPU on any of the outcome measures. In sum, neither of the confirmatory hypotheses were supported. 

Although not hypothesized a priori, MLM analyses showed a statistically significant main effect of time on both negative affect (*b* = − 0.01, *t*[167.64] = − 2.68, *p* = 0.008, semi-partial *R*^2^ = 0.041) and withdrawal symptoms (*b* = − 0.03, *t*[172.70] = − 3.28, *p* = 0.001, semi-partial *R*^2^ = 0.061), indicating a decrease in negative affect and withdrawal scores in both groups over the experimental period.

#### Exploratory Analyses

In considering the lack of support for the confirmatory hypotheses, it was noted that in terms of past 4-week FPU, the large majority of the sample reported using pornography three times a week or four times a week (61.4%), with fewer reporting five times a week or six times a week (19.3%) and once a day or more than once a day (19.3%). This appeared to be contributed to by the large proportion of female participants (64.2%) in the sample, most of whom reported past 4-week FPU of three times a week or four times a week (77.0%), with fewer reporting five times a week or six times a week (13.3%) and once a day or more than once a day (9.7%). This was in contrast to male participants, who had a roughly equal proportion of participants reporting past 4-week FPU of three times a week or four times a week (32.8%), five times a week or six times a week (31.1%) and once a day or more than once a day (36.1%). Examination of baseline characteristics of the sample by gender showed statistically significant gender differences, most notably male participants having significantly higher PPU (*F*[1,172] = 9.027, *p* = 0.003), craving (*F*[1,172] = 7.168, *p* = 0.008) and past 4-week FPU (*F*[1,172] = 51.768, *p* < 0.001) than female participants (see Table S10 in Supplementary Material for a complete summary of baseline characteristics by gender).

On the basis that abstinence effects might manifest only at higher levels of past 4-week FPU, exploratory MLM analyses were run on each outcome measure to account for potential moderating effects of past 4-week FPU. All variables in the confirmatory MLM models were retained, but with past 4-week FPU now specified as a moderator (i.e., three-way interaction [group × PPU × past 4-week FPU] or two-way interaction [group × past 4-week FPU]; see Tables S11 and S12 in Supplementary Material).^8^ Given that there were important gender differences in baseline characteristics, gender was controlled for as a covariate in these models. MLM results showed that past 4-week FPU played a significant moderating role in the craving model but not the positive affect, negative affect and withdrawal symptoms models (see Table 2).

**Fig. 3:**
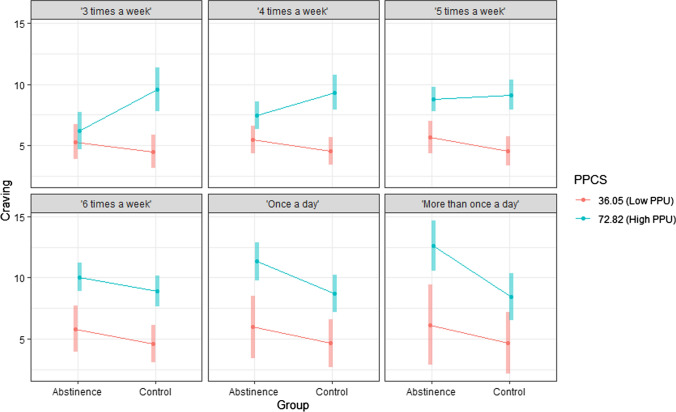
Effect of group on craving by PPU level (± 1 SD) at all six levels of past 4-week FPU

In the craving model, there was a significant main effect of group (*b* = − 7.36, *t*[160.97] = − 2.04, *p* = 0.043, semi-partial *R*^*2*^ = 0.025), a significant two-way group × PPU interaction (*b* = 0.19, *t*[161.66] = 2.93, *p* = 0.004, semi-partial *R*^*2*^ = 0.050) and a significant two-way PPU × past 4-week FPU use interaction (*b* = 0.03, *t*[159.08] = 2.46, *p* = 0.015, semi-partial *R*^*2*^ = 0.013). However, the main effect and two-way interactions were qualified by a significant three-way group × PPU × past 4-week FPU interaction (*b* = − 0.04, *t*[160.43] = − 2.30, *p* = 0.023, semi-partial *R*^*2*^ = 0.032). To probe the pattern of this three-way interaction, contrasts between abstinence and control groups (see Table S13 in Supplementary Material) were examined at combinations of either high (+ 1 SD) or low (-1 SD) values on the PPCS and six values corresponding to all six response anchors on the past 4-week FPU item (1 = “3 times a week,” 2 = “4 times a week,” 3 = “5 times a week,” 4 = “6 times a week,” 5 = “once a day” and 6 = “more than once a day”). The plot of this interaction is presented in Fig. 3.

At low levels of PPU, there were no significant differences between abstinence and control groups on craving at all levels of past 4-week FPU. However, at high levels of PPU, there were differential effects of group on craving depending on level of past 4-week FPU. More specifically, the control group had significantly higher craving than the abstinence group over the experimental period when PPU level was high and past 4-week FPU was “3 times a week” (*b* = − 3.38, *t*[163] = − 3.04, *p* = 0.016; abstinence group estimated marginal mean [EMM] = 6.19, SE = 0.77 vs. control group EMM = 9.57, SE = 0.90). On the contrary, the abstinence group had significantly higher craving than the control group over the experimental period when PPU level was high and past 4-week FPU was “once a day” (*b* = 2.65, *t*[160] = 2.65, *p* = 0.036; abstinence group EMM = 11.34, SE = 0.79 vs. control group EMM = 8.69, SE = 0.77) or when PPU level was high and past 4-week FPU was “more than once a day” (*b* = 4.16, *t*[161] = 3.17, *p* = 0.016; abstinence group EMM = 12.62, SE = 1.04 vs. control group EMM = 8.47, SE = 0.97). There were no significant differences between abstinence and control groups at high levels of PPU and when past 4-week FPU was “4 times a week,” “5 times a week” or “6 times a week.”

Finally, at the request of a reviewer, a supplementary set of exploratory MLM analyses was run on each outcome measure to rule out the possibility that abstinence effects manifested only at specific time points (e.g., in the middle or toward the end) of the 7-day period. To achieve this, all variables in the previous exploratory MLM models were retained, but with time now specified as a moderator (i.e., three-way interaction [group × PPU × time] or two-way interaction [group × time]; see Tables S14-S16 in Supplementary Material). In all models, both linear and quadratic time terms were specified, to allow for potential nonlinear trends. MLM results indicated no significant group × PPU × (linear/quadratic) time nor group × (linear/quadratic) time interactions in any of the models, suggesting that there were no significant abstinence effects on any of the outcome variables at a specific time point of the 7-day period. Overall, it is important to emphasize that all exploratory analyses in the present study should be interpreted with caution given the limited statistical power in the present study’s sample to detect three-way interaction effects if any exist.

## Discussion

The present study used a randomized controlled design to examine whether (1) negative abstinence effects potentially reflective of withdrawal-related symptoms manifest when regular pornography users (defined in the present study as having used pornography ≥ three times a week in the past four weeks) try to abstain from pornography for a 7-day period and (2) these negative abstinence effects only manifest (or manifest more strongly) for those with higher levels of PPU.

### Confirmatory Analyses

The results showed that both confirmatory hypotheses (H_1_ and H_2_) were not supported. Contrary to the first hypothesis, there were no significant main effects of group (abstinence vs. control) on craving, negative affect, positive affect or withdrawal symptoms during the experimental period, controlling for baseline scores. This indicates that when assessed prospectively in comparison with a non-abstaining control group, no evidence of withdrawal-related symptoms was found for the abstaining participants in this sample. This finding is in contrast to previous cross-sectional research relying on retrospective self-report that found 72.2% of pornography users who had at least one past pornography abstinence attempt recalled experiencing at least one withdrawal-like symptom upon cessation (Dwulit & Rzymski, 2019).

The second hypothesis that negative abstinence effects during the experimental period would only manifest (or manifest more strongly) for those with higher levels of PPU was also not supported, as there were no significant group × PPU interaction effects on all outcome measures. Nonetheless, it is important to interpret this finding in light of the fact that because this was a non-clinical sample of regular pornography users, the range of scores on the PPCS was relatively low [*M* = 54.72, SD = 18.60, with only 15.3% of the sample having PPCS scores that met or exceeded the suggested clinical cutoff of ≥ 76 in Bőthe et al. (2018)]. This finding does not rule out the possibility that in a sample with higher levels of PPU, moderating effects of PPU might be observed.

An unexpected finding was that both negative affect and withdrawal symptoms decreased over the experimental period for both abstinence and control groups. This decrease appeared to be indicative of the “downward drift” phenomenon (see Gilbert et al., 2019, p. 538), which is the tendency for participants to report progressively fewer negative affect-related symptoms when tested repeatedly, regardless of assignment to treatment or control. This phenomenon has been observed in studies of abstinence from cigarette smoking (Gilbert et al., 2002) and gaming (Evans et al., 2018) and also non-abstinence-related studies (e.g., Sharpe & Gilbert, 1998). While multiple explanations have been proposed for the phenomenon (Arrindell, 2001; Sharpe & Gilbert, 1998), one plausible explanation is that repeated testing (in the present study, the daily surveys) acts as an intervention that facilitates self-monitoring of negative affect and possibly coping mechanisms to deal with the negative affect. This finding further underscores the importance of including a non-abstaining control group in future prospective abstinence studies, as decreases in negative affective symptoms over time by abstaining participants could easily be misattributed to abstinence.

### Exploratory Analyses

Importantly, exploratory analyses provided partial support for the possibility that the null findings observed in the confirmatory analyses could have been the result of not accounting for the moderating effects of a third variable (i.e., past 4-week FPU). The majority of the sample (61.4%) reported past 4-week FPU on the lower end of the spectrum (i.e., three or four times a week), in large part contributed to by the large proportion of female participants in the sample (64.2%) most of whom also reported past 4-week FPU of three or four times a week (77.0%). Consistent with past research (Chen et al., 2018; Grubbs et al., 2019c; Weinstein et al., 2015), female participants in this sample had lower baseline rates of FPU, PPU and craving than male participants. Therefore, the possibility that abstinence effects might only manifest at higher levels of FPU was examined in exploratory models with past 4-week FPU added as a moderator and gender controlled for as a covariate.

A significant three-way group × PPU × past 4-week FPU interaction on craving was found, but past 4-week FPU did not play a significant moderating role in the negative affect, positive affect and withdrawal symptoms models. At high levels of PPU (+ 1 SD), the control group had higher craving scores than the abstinence group over the experimental period when past 4-week FPU was “three times a week,” but inversely the abstinence group had higher craving scores than the control group over the experimental period when past 4-week FPU was “once a day” or “more than once a day.” Notably, this indicates that there was an abstinence effect on craving when PPU was high, but only once past 4-week FPU reached the threshold of daily use. It is noteworthy that this effect was found even though high PPU at + 1 SD (i.e., *M* = 72.82) in this sample did not reach the clinical cutoff of ≥ 76 specified in Bőthe et al. (2018). This finding is similar to a previous cross-sectional survey of undergraduates in Canada that found an increase in addiction-related symptomatology once FPU reached the threshold of daily use (Harper & Hodgins, 2016). It is uncertain as to why the abstinence group reported significantly lower craving than the control group at high levels of PPU and past 4-week FPU of ‘three times a week,’ but one speculative explanation could be that abstaining participants below a specific threshold of FPU (i.e., three times a week) due to lower habit strength (Sirriani & Vishwanath, 2016) had enough self-regulation such that they were able to successfully implement strategies to regulate their experience of craving (e.g., prevent its occurrence or reduce its intensity whenever it did emerge) to help them achieve the abstinence goal.

There are two possible interpretations of the abstinence effect on craving under high PPU and high FPU conditions that are not necessarily mutually exclusive. First, if negative abstinence effects are interpreted within an addiction framework as withdrawal-related symptoms, then craving could potentially be a withdrawal symptom in PPU. High FPU on its own (without high PPU) may be indicative of preoccupation with pornography that may not be associated with negative life consequences (Bőthe et al., 2020a, 2020b), but the fact that craving manifested for those with daily or more FPU only when PPU was high reinforces the idea that abstinence-induced craving could be reflective of a dependency on pornography and potentially a withdrawal symptom (if a pornography withdrawal syndrome exists). This interpretation would be consistent with a finding from a recent systematic review that craving was the most common abstinence effect across multiple potential behavioral addictions (Fernandez et al., 2020). Second, craving could also have been a manifestation of sexual desire and/or arousal during the experimental period. Because higher baseline rates of FPU could have to some extent been reflective of higher sexual drive (Leonhardt et al., 2021), the fact that craving only manifested for those with high PPU when FPU was daily or more supports the sexual desire explanation. If participants’ primary sexual outlet was masturbating to pornography, then urges to use pornography could be a natural manifestation of sexual desire and/or arousal throughout the deprivation period (Castro-Calvo et al., 2022). The fact that an abstinence effect was found for craving and not the other outcome variables (i.e., positive affect, negative affect and withdrawal symptoms) further lends support to this non-pathological interpretation, because affect-related disturbances would arguably be expected to also be present if an actual withdrawal syndrome exists. At the same time, the lack of abstinence effects on these other outcome variables needs to be interpreted with caution because given the limited statistical power in the sample to detect three-way interaction effects, similar but smaller effects may not have been detected for these variables. Overall, all findings in these exploratory analyses should be interpreted with caution because of their post hoc exploratory nature and limited statistical power to detect effects of interest. While these exploratory findings are noteworthy, they should be regarded as hypothesis-generating for future studies.

### Implications

In sum, the findings of the present study have two main implications for understanding the potential manifestation of withdrawal-related symptoms during pornography abstinence. First, confirmatory analyses showed that there was no evidence of negative abstinence effects (i.e., withdrawal-related symptoms) among a sample of pornography users who were using pornography at least three times a week, and this was not dependent on level of PPU (but with the caveat that the sample had relatively low levels of PPU). These null findings are important to emphasize as they provide preliminary evidence that the average regular pornography user who uses pornography somewhat regularly (i.e., a few times a week) generally does not experience withdrawal-like symptoms while trying to abstain from pornography for a 7-day period.

Second, exploratory findings raise the possibility that negative abstinence effects might manifest only when both baseline FPU and PPU are high—a hypothesis that needs to be tested in future prospective studies. Exploratory analyses found that craving was an abstinence effect when PPU was high, but only once past 4-week FPU was at least daily or more. This tentatively suggests that craving could potentially be a PPU withdrawal symptom if a pornography withdrawal syndrome exists, but future adequately powered studies need to verify this finding a priori and rule out alternative theoretical explanations (e.g., craving being solely a manifestation of sexual arousal). While craving was the only significant abstinence effect under these conditions, it cannot be ruled out that adequately powered studies might find other abstinence effects.

Overall, it is crucial that the findings of the present study be considered in terms of its specific sample characteristics (i.e., non-clinical, majority female sample of undergraduates from a sexually conservative country, most of whom were using pornography 3–4 times a week [61.4%], had PPCS scores below the clinical cutoff of 76 [84.7%] and had no intrinsic desire to quit their pornography use [89.8%]). These findings may not generalize to clinical samples, non-clinical samples with higher FPU or PPU, predominantly male samples, samples from more sexually liberal countries or samples composed solely of pornography users intrinsically motivated to quit their pornography use. Future studies using similar prospective designs and diverse samples with varying gender ratios and levels of FPU, PPU and intrinsic motivation to abstain from pornography use are needed to replicate and extend these findings.

It is also important to consider when interpreting the present study’s findings that the study period (February–March 2021) was during the third wave of the COVID-19 pandemic in Malaysia, when movement control and social distancing measures were enforced throughout the country (Rampal & Liew, 2021; Zamri et al., 2021). This could have potentially influenced the present study’s findings in two ways. First, participants’ self-reported affective states during the study period could potentially have been impacted by elevated psychological distress levels brought about by the pandemic (Marzo et al., 2021; Necho et al., 2021). Second, participants’ sexual lives could have also been affected by the pandemic. Frequency of partnered sex among participants may have decreased due to reduced access to potential sexual partners (Herbenick et al., 2022). While some longitudinal data have shown that as a general trend, FPU and PPU did not increase over time during the pandemic, this might not necessarily have been the case for all pornography users (Grubbs et al., 2022; Koós et al., 2022). For example, there has been evidence of retrospective self-reports, particularly among male participants, of increased FPU and frequency of masturbation during the pandemic when compared to pre-pandemic frequencies (Gleason et al., 2021; Sallie et al., 2021). It is plausible that some participants in the present study may have developed an increased reliance on masturbating to pornography as a sexual outlet in the absence of opportunities for partnered sex during stay-at-home orders, which could have made abstaining from pornography more challenging than usual. In sum, it is important to take into account that the present study’s findings may not necessarily generalize to a post-pandemic setting.

### Limitations and Directions for Future Research

The present study has several limitations that need to be highlighted. First, because the study’s aim was to investigate abstinence effects irrespective of gender, there were no gender restrictions in the inclusion criteria, resulting in a sample that was close to two-thirds (64.2%) female. Compared to females, males tend to use pornography more for sexual pleasure (Bőthe et al., 2021a; Grubbs et al., 2019c) and have a stronger sex drive in general (Baumeister et al., 2001). As such, males may be more reliant on pornography as a sexual outlet than females. Therefore, it may be reasonable to speculate that males could have greater difficulty in abstaining from pornography than females even if they have similar rates of baseline FPU. However, because females had lower past 4-week FPU than males in the present sample, exploratory analyses with gender as moderator were not run because they would have been difficult to disentangle from exploratory analyses with past 4-week FPU as moderator. Future studies could consider restricting inclusion criteria to just male participants to examine whether abstinence effects manifest in a male-only sample or run adequately powered studies with both genders included (but with narrower inclusion criteria for baseline FPU [e.g., ≥ six times a week]) to investigate potential varying abstinence effects by gender, if any. Differing contexts of pornography use across genders (e.g., women in relationships are more likely than men to use pornography primarily or only with their partner; Carroll et al., 2017) could also be accounted for as a potential moderator of effects in future studies.

Second, nearly half (45.4%) of participants in the abstinence group reported using pornography at least once during the experimental period, which could have led to a mitigation in the frequency and/or intensity of any withdrawal-related symptoms experienced for these participants. While excluding participants who lapse from analyses may introduce bias because these participants are likely to experience the greatest amount of withdrawal in the first place (Hughes, 2007b; Piasecki et al., 2003a, 2003b; Shiffman et al., 2004), it is important to keep in mind that withdrawal-related symptom scores of the abstinence group as a whole may have been influenced by the considerable proportion of participants who did lapse in the present study.

Third, because the focus of the present study was to examine effects of abstinence from pornography specifically, participants in the abstinence group were allowed to masturbate without pornography or engage in non-pornography-related sexual activity. An advantage of allowing non-pornography-related sexual outlets is that potential manifestations of withdrawal-like symptoms can be more clearly attributed to a dependency on pornography use instead of dependency on masturbation or sexual activity (e.g., if withdrawal-like symptoms persist even after masturbating without pornography). However, a drawback of this is that it may not resemble many real-world abstinence attempts, given that many individuals with PPU may decide to abstain from pornography and masturbation or abstain from sexual activity altogether as a short-term recovery strategy (Fernandez et al., 2021). Future studies could consider modifying abstinence instructions so that they also include masturbation or sexual activity altogether.

Fourth, while the experimental design used in the present study contributed to rigorous internal validity due to randomization and use of a control group, experimental designs are inherently limited in the extent to which the studies’ results are generalizable to the wider population (because of the use of a relatively small, homogenous sample) and applicable to real-world situations (because of the emphasis on controlled conditions that prioritize internal validity over ecological validity). Importantly, abstinence in the present study may not be representative of real-world abstinence attempts because participants knew that abstinence would only last for a temporary 7-day period, they could lapse without consequence, and they could terminate their participation at any moment. Non-experimental longitudinal studies of intrinsic abstinence attempts are needed to supplement data provided by studies that experimentally manipulate abstinence.

Fifth, because it is plausible that a pornography withdrawal syndrome, akin to the withdrawal syndromes of some substances (cf. Hughes et al., 1994), might only begin or worsen after a significant period of abstinence (e.g., one or two weeks), the fairly brief duration of the present study (i.e., 7 days) meant that any potential withdrawal symptoms that might have emerged beyond an initial 7-day period could not be captured. Future studies could consider increasing the duration of the abstinence period to investigate this possibility.

Sixth, the outcome measures used to assess potential pornography withdrawal-related symptoms in the present study were a limitation because they were modified from existing scales constructed to assess alcohol craving (Flannery et al., 1999), smoking withdrawal symptoms (Welsch et al., 1999) and general affect (Thompson, 2007). Measures of pornography-specific withdrawal-related symptoms need to be developed and validated for use in future abstinence studies.

Seventh, despite having a significantly shorter recall period compared to a single end-of-week assessment, end-of-day surveys still rely on retrospection and as such remain susceptible to some recall bias (Newman & Stone, 2019). Research comparing aggregated momentary affect ratings throughout the day to retrospective end-of-day affect ratings demonstrates that end-of-day ratings of negative affect tend to be slightly biased toward peak and recent affect (Neubauer et al., 2020). Future studies can use ecological momentary assessment (EMA) instead of daily surveys for greater sensitivity to fluctuations of affect throughout the day.

Eighth, because the baseline measures for all outcome variables had a bigger recall period (past 7 days) compared to the daily measures (past day), changes in daily scores relative to baseline could not be examined. Incorporating a pre-intervention period where baseline data are also collected using the same daily surveys would allow for standardization of measures and examination of changes from baseline.

Finally, the present study did not account for the type or genre of pornography that participants typically used. It is possible that specific types of pornography (e.g., non-mainstream content; Hald et al., 2018) or greater variability of pornography content consumed (Lewczuk et al., 2021) may be associated with increased difficulty in abstaining from pornography use and can be accounted for in future abstinence studies.

### Conclusion

Current understanding of the potential manifestation of withdrawal-related symptoms during abstinence from pornography is still in its infancy. The present study is the first to prospectively examine the manifestation of withdrawal-related symptoms in abstaining participants in comparison to a non-abstaining control group, in contrast to previous research that has relied on retrospective self-report of perceived abstinence effects. The present study contributes preliminary data that represent a first step toward understanding whether and under what conditions withdrawal-related symptoms may (or may not) manifest when regular pornography users attempt to abstain from pornography use for a 7-day period. More prospective data across diverse samples are needed to verify and expand upon the findings of the present study.

## Supplementary Information

Below is the link to the electronic supplementary material.

Supplementary file1 (DOCX 118 KB)

## Footnotes

1 Primary reasons cited include: (1) withdrawal not being necessary for the diagnosis of addiction more broadly (as the cessation of some substances does not produce a withdrawal syndrome—Starcevic, 2016) and (2) methodological weaknesses in the assessment of withdrawal in behavioral addictions (e.g., relying on individuals’ subjective complaints of feeling irritable instead of psychophysiological measurement) has meant that the evidence base supporting the existence of behavioral addiction withdrawal syndromes has been argued to be weak (Kardefelt-Winther et al., 2017; Pies, 2009; van Rooij & Prause, 2014).

2 Prospective participants were informed about the nature of the random assignment from the outset because a willingness to attempt abstinence from pornography if assigned to the abstinence group was necessary for participation. While this may have led to demand characteristics, it was deemed unethical to withhold this information from prospective participants.

3 Because the control group had been randomly allocated one or two participants more than the abstinence group in all three surveys corresponding to the three study sessions (i.e., February session [67 vs. 65], first March session [16 vs. 15], second March session [7 vs. 6]), this added up to a slightly higher total number of participants in the control group than the abstinence group (90 vs. 86).

4 The PACS has also been adapted as a measure of gaming withdrawal symptomatology in previous gaming abstinence studies (Evans et al., 2018; Katpsis et al., 2016a).

5 Specific models that had non-convergence issues and how these were addressed are detailed in Tables S6, S11 and S15.

6 Confirmatory analyses refer to analyses that tested the present study’s a priori hypotheses, while exploratory analyses refer to analyses that did not test a priori hypotheses but were conducted in a post-hoc, exploratory capacity.

7 Because the baseline score of each outcome measure represented a retrospective estimation of how participants felt in general over the past seven days, whether daily scores increased or decreased relative to baseline was not examined. The tendency for individuals to overestimate the intensity of positive and negative affect when recalling past affect is a robust finding in the literature (Ben-Zeev et al., 2009; Kardum & Daskijević, 2001; Thomas & Diener, 1990). Therefore, the baseline scores would be expected to be inflated relative to the daily scores and as such were only controlled for as covariates.

8 All exploratory models in the present study followed the same backward elimination procedure as the confirmatory models, where more complex models were compared to reduced models until the best-fitting model was found for each outcome measure.
