# Supplementary material for: Effects of a 7-Day Pornography Abstinence Period on Withdrawal-Related Symptoms in Regular Pornography Users: A Randomized Controlled Study
Source: Arch Sex Behav. 2023 Jan 18;52(4):1819–40. doi: 10.1007/s10508-022-02519-w (PMC9847461; doi:10.1007/s10508-022-02519-w)
Supplement: Supplementary file 1 — Supplementary file1 (DOCX 118 KB) [file 10508_2022_2519_MOESM1_ESM.docx]

SUPPLEMENTARY MATERIAL FOR:

**Effects of a seven-day pornography abstinence period on withdrawal-related symptoms in regular pornography users: A randomized controlled study**

| **Table of contents** | |
| --- | --- |
| Table/appendix | Page number |
| Table S1: Baseline characteristics of the sample | 2 |
| Appendix A: Full instructions provided to the abstinence and control groups | 5 |
| Appendix B: Measures used in the present study | 6 |
| Appendix C: Detailed summary of validity of outcome measures | 11 |
| Table S2: Fit indices from confirmatory factor analyses (CFA) of the outcome measures | 13 |
| Table S3: Standardized factor loadings from confirmatory factor analyses (CFA) of the outcome measures | 14 |
| Table S4: Inter-factor correlations from confirmatory factor analyses (CFA) of I-PANAS-SF and 14-item WSWS adapted for pornography use | 15 |
| Table S5: Associations between outcome measures and pornography use variables at baseline | 16 |
| Table S6: Multilevel model results for manipulation check analyses | 17 |
| Table S7: Means and standard deviations of all study variables for abstinence and control groups | 18 |
| Table S8: Model fit statistics of multilevel models in confirmatory analyses | 20 |
| Table S9: Model comparisons of multilevel models in confirmatory analyses | 22 |
| Table S10: Baseline characteristics of the sample by gender | 23 |
| Table S11: Model fit statistics of multilevel models in exploratory analyses with past four-week FPU as moderator | 25 |
| Table S12: Model comparisons of multilevel models in exploratory analyses with past four-week FPU as moderator | 27 |
| Table S13: Contrasts between abstinence and control groups at combinations of high (+1 SD) or low (-1 SD) PPU and all six levels of past four-week FPU | 28 |
| Table S14: Multilevel model results for all outcome variables in exploratory analyses with time as moderator | 29 |
| Table S15: Model fit statistics of multilevel models in exploratory analyses with time as moderator | 30 |
| Table S16: Model comparisons of multilevel models in exploratory analyses with time as moderator | 32 |
| References used in this supplementary document | 33 |

**Table S1**

*Baseline characteristics of the sample*

| Characteristic | Abstinence group  (*n* = 86) | Control group  (*n* = 90) | Total  (*N* = 176) |
| --- | --- | --- | --- |
| *Age, M (SD)* | 21.17 (1.26) | 21.57 (1.69) | 21.38 (1.50) |
| *Gender,* *n* (%) |  |  |  |
| Male | 30 (34.88) | 31 (34.44) | 61 (34.66) |
| Female | 55 (63.95) | 58 (64.44) | 113 (64.20) |
| Agender | 1 (1.16) | 1 (1.11) | 2 (1.14) |
| *Sexual orientation*, *n* (%) |  |  |  |
| Heterosexual | 67 (77.9) | 69 (76.67) | 136 (77.27) |
| Homosexual | 3 (3.49) | 5 (5.56) | 8 (4.55) |
| Bisexual | 11 (12.79) | 11 (12.22) | 22 (12.50) |
| Other (combined^a^) | 5 (5.81) | 5 (5.56) | 10 (5.68) |
| *Relationship status, n* (%) |  |  |  |
| Single, not in a committed relationship | 45 (52.33) | 47 (52.22) | 92 (52.27) |
| In a committed relationship | 40 (46.51) | 43 (47.78) | 83 (47.16) |
| Married | 1 (1.16) | 0 (0.00) | 1 (0.57) |
| *Nationality, n* (%) |  |  |  |
| Malaysian | 82 (95.35) | 81 (90.00) | 163 (92.61) |
| Indonesian | 2 (2.33) | 4 (4.44) | 6 (3.41) |
| Other (combined^b^) | 2 (2.33) | 5 (5.56) | 7 (3.98) |
| *Past four-week frequency of pornography use*, *n* (%) |  |  |  |
| 3 times a week on average | 29 (33.72) | 33 (36.67) | 62 (35.23) |
| 4 times a week on average | 23 (26.74) | 23 (25.56) | 46 (26.14) |
| 5 times a week on average | 13 (15.12) | 6 (6.67) | 19 (10.80) |
| 6 times a week on average | 6 (6.98) | 9 (10.00) | 15 (8.52) |
| Once a day on average | 12 (13.95) | 11 (12.22) | 23 (13.07) |
| More than once a day on average | 3 (3.49) | 8 (8.89) | 11 (6.25) |
| *Past four-week average duration of pornography use per session*, *n* (%) |  |  |  |
| 5 minutes or less | 7 (8.14) | 6 (6.67) | 13 (7.39) |
| 6 to 10 minutes | 23 (26.74) | 20 (22.22) | 43 (24.43) |
| 11 to 20 minutes | 22 (25.58) | 27 (30.00) | 49 (27.84) |
| 21 to 30 minutes | 17 (19.77) | 18 (20.00) | 35 (19.89) |
| 31 to 45 minutes | 8 (9.30) | 13 (14.44) | 21 (11.93) |
| 46 to 60 minutes | 5 (5.81) | 3 (3.33) | 8 (4.55) |
| 1 to 1.5 hours | 3 (3.49) | 3 (3.33) | 6 (3.41) |
| 1.5 to 2 hours | 1 (1.16) | 0 (0.00) | 1 (0.57) |
| *Past four-week frequency of masturbation without pornography*, *n* (%) |  |  |  |
| Never | 40 (46.51) | 39 (43.33) | 79 (44.89) |
| 3 times or less in total | 27 (31.40) | 30 (33.33) | 57 (32.39) |
| Once a week on average | 12 (13.95) | 6 (6.67) | 18 (10.23) |
| 2-3 times a week on average | 7 (8.14) | 7 (7.78) | 14 (7.95) |
| 4-6 times a week on average | 0 (0.00) | 5 (5.56) | 5 (2.84) |
| 7 or more times a week on average | 0 (0.00) | 3 (3.33) | 3 (1.70) |
| *Past four-week percentage of pornography sessions accompanied by masturbation* (range 0-100), *M (SD)* | 70.81 (32.09) | 67.80 (34.65) | 69.27 (33.36) |
| *Past seven-day total frequency of pornography use, M (SD)* | 5.93 (4.49) | 5.42 (2.96) | 5.67 (3.78) |
| *Past seven-day frequency of pornography use without masturbation, M (SD)* | 1.56 (3.77) | 1.69 (2.37) | 1.62 (3.12) |
| *Past seven-day frequency of pornography use with masturbation, M (SD)* | 4.37 (3.63) | 3.73 (3.02) | 4.05 (3.34) |
| *Past seven-day duration of pornography use in minutes, M (SD)* | 108.88 (100.71) | 110.60 (109.75) | 109.76 (105.13) |
| *Problematic pornography use* (range 18-126), *M (SD*) | 57.69 (20.00) | 51.88 (1.68) | 54.72 (18.60) |
| PPCS score < 76 (clinical cutoff), *n* (%) | 69 (80.23) | 80 (88.89) | 149 (84.66) |
| PPCS score ≥ 76 (clinical cutoff), *n* (%) | 17 (19.77) | 10 (11.11) | 27 (15.34) |
| *Moral disapproval of pornography* (range 1-7), *M (SD)* | 2.83 (1.68) | 2.68 (1.51) | 2.75 (1.59) |
| *Intrinsic desire to quit or reduce pornography use, n* (%) |  |  |  |
| Reduce | 37 (43.02) | 49 (54.44) | 86 (48.86) |
| Quit | 11 (12.79) | 7 (7.78) | 18 (10.23) |
| No desire to reduce or quit | 38 (44.19) | 34 (37.78) | 72 (40.91) |
| *Abstinence effort (range 0-10), M (SD)* | 1.06 (2.48) | 1.19 (2.51) | 1.13 (2.49) |
| *Craving* (range 0-24), *M (SD)* | 12.55 (3.40) | 11.51 (3.93) | 12.02 (3.70) |
| *Negative affect* (range 5-25), *M (SD)* | 12.50 (4.14) | 12.24 (4.40) | 12.37 (4.26) |
| *Positive affect* (range 5-25), *M (SD)* | 14.41 (3.47) | 14.39 (4.00) | 14.40 (3.74) |
| *Withdrawal symptoms* (range 0-4), *M (SD)* | 2.15 (0.66) | 2.13 (0.74) | 2.14 (0.70) |

*Note*. ^a^Pansexual, asexual, other, prefer not to say; ^b^Bruneian, Chinese, Iranian, Maldivian. *M* = mean, *SD* = standard deviation.

**Appendix A: Full instructions provided to the abstinence and control groups**

*Instructions provided to abstinence group*

Dear participant,

**You have been randomly assigned to the ‘abstinence’ group.**

*Instructions*

Please try your best to abstain from watching any pornography for the next 7 days. The seven-day period starts from 5.00 am on Tuesday, [date] and ends at 5.00 am on Tuesday, [date].

‘Pornography’ is defined here as follows:

“any sexually explicit films, video clips or pictures which intends to sexually arouse the viewer; this may be seen on the Internet, in a magazine, in a book, or on television.”

Please note that successfully achieving complete abstinence over the 7 days is not required for your participation in the present study to be considered valid – what matters is that you are trying your best to not watch any pornography during this time. You will also be free to masturbate without using pornography or engage in any other non-pornography related sexual activity (e.g., have sexual intercourse) during the 7 days – the instruction is simply to try your best to not watch pornography. If you do happen to watch pornography during this time, you will be asked to report this honestly during the daily diary surveys, and this will not have an effect on any compensation you may be entitled to receive.

*Instructions provided to control group*

Dear participant,

**You have been randomly assigned to the ‘no abstinence’ group.**

*Instructions*

You are free to watch pornography as usual for the duration of this study (i.e., the next 7 days).

**Appendix B: Measures used in the present study**

**Outcome measures (baseline and daily)**

*Craving (baseline and daily)*

| Please read each question carefully and select the option that best describes your craving/desire to watch pornography during the past 7 days/since waking up today^a^. | |
| --- | --- |
| 1. During the past 7 days/since waking up today, *how often* have you thought about watching pornography or about how good watching pornography would make you feel? | |
| ○ | 0 – Never |
| ○ | 1 – Rarely |
| ○ | 2 – Occasionally |
| ○ | 3 – Sometimes |
| ○ | 4 – Often |
| ○ | 5 – Most of the time |
| ○ | 6 – Nearly all of the time |
|  |  |
| 1. At its most severe point, *how strong* was your craving to watch pornography during the past 7 days/since waking up today? | |
| ○ | 0 – None at all |
| ○ | 1 – Slight (very mild) urge |
| ○ | 2 – Mild urge |
| ○ | 3 – Moderate urge |
| ○ | 4 – Strong urge but easily controlled |
| ○ | 5 – Strong urge and difficult to control |
| ○ | 6 – Strong urge and uncontrollable |
|  |  |
| 1. During the past 7 days/since waking up today, *how difficult would it have been* to resist watching pornography if you knew you had the opportunity to watch pornography? | |
| ○ | 0 – Not difficult at all |
| ○ | 1 – Very mildly difficult |
| ○ | 2 – Mildly difficult |
| ○ | 3 – Moderately difficult |
| ○ | 4 – Very difficult |
| ○ | 5 – Extremely difficult |
| ○ | 6 – Would not be able to resist |
|  |  |
| 1. Keeping in mind your responses to the previous questions, please rate your *overall average craving to watch pornography* during the past 7 days/since waking up today. | |
| ○ | 0 – Never thought about watching pornography and never had the urge to watch |
| ○ | 1 – Rarely thought about watching pornography and rarely had the urge to watch |
| ○ | 2 – Occasionally thought about watching pornography and occasionally had the urge to watch |
| ○ | 3 – Sometimes thought about watching pornography and sometimes had the urge to watch |
| ○ | 4 – Often thought about watching pornography and often had the urge to watch |
| ○ | 5 – Thought about watching pornography most of the time and had the urge to watch most of the time |
| ○ | 6 – Thought about watching pornography nearly all of the time and had the urge to watch nearly all of the time |

^a^Time frame for baseline survey = during the past 7 days; time frame for daily survey = since waking up today.

*Withdrawal symptoms (baseline and daily)*

| Please answer the following questions based on how you have felt or what you have noticed *over the past 7 days/since waking up today^a^.* Answer based on how you felt in general during this time. | | | | | |
| --- | --- | --- | --- | --- | --- |
|  | Strongly disagree  0 | Disagree  1 | Feel neutral  2 | Agree  3 | Strongly  agree  4 |
| 1. I have been tense or anxious. | ○ | ○ | ○ | ○ | ○ |
| 2. My level of concentration is excellent*. | ○ | ○ | ○ | ○ | ○ |
| 3. I have felt impatient. | ○ | ○ | ○ | ○ | ○ |
| 4. I have felt upbeat and optimistic*. | ○ | ○ | ○ | ○ | ○ |
| 5. I have found myself worrying about my problems. | ○ | ○ | ○ | ○ | ○ |
| 6. I have felt calm lately*. | ○ | ○ | ○ | ○ | ○ |
| 7. I have felt sad or depressed. | ○ | ○ | ○ | ○ | ○ |
| 8. I have been irritable, easily angered. | ○ | ○ | ○ | ○ | ○ |
| 9. I have been bothered by negative moods such as anger, frustration and irritability. | ○ | ○ | ○ | ○ | ○ |
| 10. I have felt frustrated. | ○ | ○ | ○ | ○ | ○ |
| 11. I have felt hopeless and discouraged. | ○ | ○ | ○ | ○ | ○ |
| 12. It is hard to pay attention to things. | ○ | ○ | ○ | ○ | ○ |
| 13. I have felt happy and content*. | ○ | ○ | ○ | ○ | ○ |
| 14. It has been difficult to think clearly. | ○ | ○ | ○ | ○ | ○ |

^a^Time frame for baseline survey = over the past 7 days; time frame for daily survey = since waking up today. *These items are reversed scored.

*Positive and negative affect (baseline and daily)*

| Indicate to what extent you have felt this way *during the past 7 days/since waking up today^a^*. | | | | | |
| --- | --- | --- | --- | --- | --- |
|  | Very slightly or not at all  1 | A little  2 | Moderately  3 | Quite a bit  4 | Extremely  5 |
| Upset | ○ | ○ | ○ | ○ | ○ |
| Hostile | ○ | ○ | ○ | ○ | ○ |
| Alert | ○ | ○ | ○ | ○ | ○ |
| Ashamed | ○ | ○ | ○ | ○ | ○ |
| Inspired | ○ | ○ | ○ | ○ | ○ |
| Nervous | ○ | ○ | ○ | ○ | ○ |
| Determined | ○ | ○ | ○ | ○ | ○ |
| Attentive | ○ | ○ | ○ | ○ | ○ |
| Afraid | ○ | ○ | ○ | ○ | ○ |
| Active | ○ | ○ | ○ | ○ | ○ |

^a^Time frame for baseline survey = during the past 7 days; time frame for daily survey = since waking up today.

**Other baseline measures**

*Past four week frequency of pornography use*

| In the past 4 weeks, how often did you watch pornography, on average? | |
| --- | --- |
| ○ | 3 times a week on average |
| ○ | 4 times a week on average |
| ○ | 5 times a week on average |
| ○ | 6 times a week on average |
| ○ | Once a day on average |
| ○ | More than once a day on average |

*Past four-week frequency of masturbation without pornography*

| In the past 4 weeks, how often did you masturbate without watching pornography? | |
| --- | --- |
| ○ | Never |
| ○ | 3 times or less in total |
| ○ | About once a week on average |
| ○ | About 2-3 times a week on average |
| ○ | About 4-6 times a week on average |
| ○ | About 7 or more times a week on average |

*Past four-week duration of pornography use per session*

| In the past 4 weeks, when you watched pornography, how much time (on average) did you spend watching it PER SESSION? | |
| --- | --- |
| ○ | 5 minutes or less |
| ○ | 6 to 10 minutes |
| ○ | 11 to 20 minutes |
| ○ | 21 to 30 minutes |
| ○ | 31 to 45 minutes |
| ○ | 46 to 60 minutes |
| ○ | 1 to 1.5 hours |
| ○ | 1.5 to 2 hours |
| ○ | 2 to 3 hours |
| ○ | 3 hours or more |

*Past four-week percentage of pornography sessions accompanied by masturbation*

| In the past 4 weeks, roughly how many % of all your pornography watching sessions was accompanied by masturbation? | |
| --- | --- |
| % of pornography watching sessions accompanied by masturbation | 0 10 20 30 40 50 60 70 80 90 100 |

*Past seven-day frequency of pornography use*

| In the past 7 days, how many times in total did you watch pornography? (enter as number) |  | times |
| --- | --- | --- |
| Out of the ___ times you watched pornography in the past 7 days, please enter in the boxes below how many times you (1) watched pornography without masturbating, and (2) watched pornography while masturbating. | | |
| Watched pornography without masturbating |  | times |
| Watched pornography while masturbating |  | times |

*Past seven-day duration of pornography use*

| In the past 7 days, how much time in total (in minutes) did you spend watching pornography?  e.g., if you watched pornography for 20 minutes on Tuesday, 40 minutes on Thursday, and 15 minutes on Saturday, this would be 75 minutes in total. |  | minutes |
| --- | --- | --- |

*Past seven-day abstinence effort*

| To what extent were you trying your best to abstain from pornography in the past 7 days, on a scale of 0 (I did not try at all) to 10 (I tried my best)? | | | | | | | | | | |
| --- | --- | --- | --- | --- | --- | --- | --- | --- | --- | --- |
| I did not try at all |  |  |  |  |  |  |  |  |  | I tried my best |
| 0 | 1 | 2 | 3 | 4 | 5 | 6 | 7 | 8 | 9 | 10 |

*Intrinsic desire to quit or reduce pornography use*

| Please choose which statement below best describes you, at this point in time/right now: | |
| --- | --- |
| ○ | I want to REDUCE my pornography use, but I don’t want to completely quit/stop using pornography |
| ○ | I want to COMPLETELY QUIT/STOP using pornography |
| ○ | I have no desire to reduce or completely quit/stop using pornography |

*Moral disapproval of pornography*

| Please rate your agreement with the following statement:  I believe that pornography use is morally wrong. | |
| --- | --- |
| ○ | Strongly disagree |
| ○ | Disagree |
| ○ | Somewhat disagree |
| ○ | Neither agree nor disagree |
| ○ | Somewhat agree |
| ○ | Agree |
| ○ | Strongly agree |

*Problematic pornography use*

| Please think back to the past six months and indicate on the following 7-point scale how often or to what extent the statements  apply to you. There is no right or wrong answer. Please indicate the answer that most applies to you. | | | | | | | |
| --- | --- | --- | --- | --- | --- | --- | --- |
|  | Never  1 | Rarely  2 | Occasionally  3 | Sometimes  4 | Often  5 | Very often  6 | All the time  7 |
| 1. I felt that porn is an important part of my life | ○ | ○ | ○ | ○ | ○ | ○ | ○ |
| 2. I used porn to restore the tranquility of my feelings | ○ | ○ | ○ | ○ | ○ | ○ | ○ |
| 3. I felt porn caused problems in my sexual life | ○ | ○ | ○ | ○ | ○ | ○ | ○ |
| 4. I felt that I had to watch more and more porn for satisfaction | ○ | ○ | ○ | ○ | ○ | ○ | ○ |
| 5. I unsuccessfully tried to reduce the amount of porn I watch | ○ | ○ | ○ | ○ | ○ | ○ | ○ |
| 6. I became stressed when something prevented me from watching porn | ○ | ○ | ○ | ○ | ○ | ○ | ○ |
| 7. I thought about how good it would be to watch porn | ○ | ○ | ○ | ○ | ○ | ○ | ○ |
| 8. Watching porn got rid of my negative feelings | ○ | ○ | ○ | ○ | ○ | ○ | ○ |
| 9. Watching porn prevented me from bringing out the best in me | ○ | ○ | ○ | ○ | ○ | ○ | ○ |
| 10. I felt that I needed more and more porn in order to satisfy my needs | ○ | ○ | ○ | ○ | ○ | ○ | ○ |
| 11. When I vowed not to watch porn anymore, I could only do it for a short period of time | ○ | ○ | ○ | ○ | ○ | ○ | ○ |
| 12. I became agitated when I was unable to watch porn | ○ | ○ | ○ | ○ | ○ | ○ | ○ |
| 13. I continually planned when to watch porn | ○ | ○ | ○ | ○ | ○ | ○ | ○ |
| 14. I released my tension by watching porn | ○ | ○ | ○ | ○ | ○ | ○ | ○ |
| 15. I neglected other leisure activities as a result of watching porn | ○ | ○ | ○ | ○ | ○ | ○ | ○ |
| 16. I gradually watched more “extreme” porn, because the porn I watched before was less satisfying | ○ | ○ | ○ | ○ | ○ | ○ | ○ |
| 17. I resisted watching porn for only a little while before I relapsed | ○ | ○ | ○ | ○ | ○ | ○ | ○ |
| 18. I missed porn greatly when I didn’t watch it for a while | ○ | ○ | ○ | ○ | ○ | ○ | ○ |

**Other daily measures**

*Daily frequency of pornography use*

| After completing last night’s survey and before going to bed, how many times did you: | | |
| --- | --- | --- |
| Watched pornography without masturbating |  | times |
| Watched pornography while masturbating |  | times |
| Since waking up today, how many times did you: | | |
| Watched pornography without masturbating |  | times |
| Watched pornography while masturbating |  | times |

*Daily duration of porn use*

| After completing last night’s survey and before going to bed, how much time in total did you spend watching pornography? |  | minutes |
| --- | --- | --- |
| Since waking up today, how much time in total did you spend watching pornography? |  | minutes |

*Daily frequency of masturbation without pornography*

| After completing last night’s survey and before going to bed, how many times did you: | | |
| --- | --- | --- |
| Masturbate without watching pornography |  | times |
| Since waking up today, how many times did you: | | |
| Masturbate without watching pornography |  | times |

*Daily frequency of alternative sexual activity*

| After completing last night’s survey and before going to bed, how many times did you: | | |
| --- | --- | --- |
| Engage in any sexual activity OTHER THAN watching pornography or masturbating (e.g., oral sex, intercourse, etc.) |  | times |
| Since waking up today, how many times did you: | | |
| Engage in any sexual activity OTHER THAN watching pornography or masturbating (e.g., oral sex, intercourse, etc.) |  | times |

*Daily abstinence effort*

| Since waking up today, to what extent were you trying your best to abstain from pornography, on a scale of 0 (I did not try at all) to 10 (I tried my best)? | | | | | | | | | | |
| --- | --- | --- | --- | --- | --- | --- | --- | --- | --- | --- |
| I did not try at all |  |  |  |  |  |  |  |  |  | I tried my best |
| 0 | 1 | 2 | 3 | 4 | 5 | 6 | 7 | 8 | 9 | 10 |

**Appendix C: Detailed summary of validity of outcome measures**

***Structural validity of outcome measures***

Confirmatory factor analysis (CFA) using the weighted least squares mean- and variance-adjusted (WLSMV) estimator from the R package *lavaan* (Roseel, 2012) was conducted on each baseline outcome measure to examine their factor structure in the present sample. Fit indices are presented in Table S2, standardized factor loadings are presented in Table S3, and inter-factor correlations are presented in Table S4.

All three outcome measures demonstrated acceptable structural validity (four-item PACS adapted for pornography use: CFI = .992, TLI = .975, RMSEA = .121; I-PANAS-SF: CFI = .958, TLI = .945, RMSEA = .080; 14-item WSWS adapted for pornography use: CFI = .935, TLI = 917, RMSEA = .117). While RMSEA values were greater than .80 for both the 4-item PACS adapted for pornography use and 14-item WSWS adapted for pornography use, RMSEA values need to be interpreted cautiously because of the small sample size and in the case of the four-item PACS adapted for pornography use, small degrees of freedom (Hu & Bentler, 1999; Kenny et al., 2015), and multiple fit indices need to be considered when evaluating a model’s adequacy (Mueller & Hancock, 2010). Despite high RMSEA values, CFI values and TLI values indicated that both models had an acceptable fit to the data and all factors appeared to be well-defined by strong factor loadings.

***Construct validity of outcome measures***

Associations of the outcome measures with each other and with pornography use variables at baseline (see Table S5) were examined to assess their convergent and discriminant validity. Craving showed a strong, positive association with PPU (*r* = .73, *p*<.001), weak-to-moderate positive associations with past 7-day FPU (*r* = .21, *p* = .006) and past 4-week FPU (*r* = .35, *p*<.001), and a weak, positive association with withdrawal symptoms (*r* = .16, *p* = .030). In addition, withdrawal symptoms showed a strong, positive association with negative affect (*r* = .70, *p*<.001) and a strong, negative association with positive affect (*r* = .51, *p*<.001), but was unrelated to past seven-day FPU, past four-week FPU, and PPU. Finally, negative affect showed a weak, positive association with PPU (*r* = .16, *p* = .039), but positive affect was unrelated to PPU. Both negative affect and positive affect were unrelated to craving, past seven-day FPU and past four-week FPU. In sum, all outcome measures appeared to demonstrate evidence of convergent and discriminant validity.

**Table S2**

*Fit indices from confirmatory factor analyses (CFA) of the outcome measures*

| Model | χ^2^ (*df*) | CFI | TLI | RMSEA | 90% CI |
| --- | --- | --- | --- | --- | --- |
| Four-item PACS adapted for pornography use (one-factor; Flannery et al., 1999) | 7.143* (2) | .992 | .975 | .121 | .034-.223 |
| I-PANAS-SF (two-factor first-order; Thompson, 2007) | 72.285**(34) | .958 | .945 | .080 | .054-.106 |
| 14-item WSWS adapted for pornography use (four-factor first-order; Welsch et al., 1999) | 241.737**(71) | .935 | .917 | .117 | .101-.134 |

*Note*. *df* = degrees of freedom; CFI = comparative fix index; TLI = Tucker-Lewis Index; RMSEA = root-mean-square error of approximation; 90% CI = 90% confidence interval of RMSEA; PACS = Penn Alcohol Craving Scale; I-PANAS-SF = International Positive and Negative Affect Schedule-Short Form; WSWS = Wisconsin Smoking Withdrawal Scale. *p<.05, ** p<.001.

**Table S3**

*Standardized factor loadings from confirmatory factor analyses (CFA) of the outcome measures*

| **Items** | **Factor loadings** |
| --- | --- |
| **4-item PACS adapted for pornography use** |  |
| 1. During the past 7 days, how often have you thought about watching pornography or about how good watching pornography would make you feel? | 0.706 |
| 2. At its most severe point, how strong was your craving to watch pornography during the past 7 days? | 0.731 |
| 3. During the past 7 days, how difficult would it have been to resist watching pornography if you knew you had the opportunity to watch pornography? | 0.796 |
| 4. Keeping in mind your responses to the previous questions, please rate your overall average craving to watch pornography during the past 7 days. | 0.855 |
| **I-PANAS-SF** |  |
| *Negative affect* |  |
| 1. Upset | 0.891 |
| 2. Hostile | 0.571 |
| 4. Ashamed | 0.550 |
| 6. Nervous | 0.603 |
| 9. Afraid | 0.762 |
| *Positive affect* |  |
| 3. Alert | 0.438 |
| 5. Inspired | 0.771 |
| 7. Determined | 0.770 |
| 8. Attentive | 0.755 |
| 10. Active | 0.610 |
| **14-item WSWS adapted for pornography use** |  |
| *Anxiety* |  |
| 1. I have been tense or anxious. | 0.692 |
| 3. I have felt impatient. | 0.625 |
| 5. I have found myself worrying about my problems. | 0.705 |
| 6. I have felt calm lately*. | 0.696 |
| *Concentration* |  |
| 2. My level of concentration is excellent*. | 0.653 |
| 12. It is hard to pay attention to things. | 0.820 |
| 14. It has been difficult to think clearly. | 0.837 |
| *Sadness* |  |
| 4. I have felt upbeat and optimistic*. | 0.609 |
| 7. I have felt sad or depressed. | 0.809 |
| 11. I have felt hopeless and discouraged. | 0.775 |
| 13. I have felt happy and content*. | 0.653 |
| *Anger* |  |
| 8. I have been irritable, easily angered. | 0.787 |
| 9. I have been bothered by negative moods such as anger, frustration and irritability. | 0.868 |
| 10. I have felt frustrated. | 0.925 |

*Note*. *These items are reverse scored. PACS = Penn Alcohol Craving Scale; I-PANAS-SF = International Positive and Negative Affect Schedule-Short Form; WSWS = Wisconsin Smoking Withdrawal Scale.. All factor loadings are standardized and statistically significant at *p* <.001.

**Table S4**

*Inter-factor correlations from confirmatory factor analyses (CFA) of I-PANAS-SF and 14-item WSWS adapted for pornography use*

| **I-PANAS-SF** | 1. | 2. |  |  |
| --- | --- | --- | --- | --- |
| 1. Positive affect | - |  |  |  |
| 2. Negative affect | -.19* | - |  |  |
| **14-item WSWS adapted for pornography use** | 1. | 2. | 3. | 4. |
| 1. Anxiety | - |  |  |  |
| 2. Concentration | .762** | - |  |  |
| 3. Sadness | .895** | .703** | - |  |
| 4. Anger | .890** | .431** | .838** | - |

*Note*. I-PANAS-SF = International Positive and Negative Affect Schedule-Short Form; WSWS = Wisconsin Smoking Withdrawal Scale. *p<.05, **p<.001.

**Table S5**

*Associations between outcome measures and pornography use variables at baseline*

|  | 1. | 2. | 3. | 4. | 5. | 6. | 7. |
| --- | --- | --- | --- | --- | --- | --- | --- |
| 1. Craving^a^ | - |  |  |  |  |  |  |
| 2. Positive affect^a^ | -.10 | - |  |  |  |  |  |
| 3. Negative affect^a^ | .12 | -.13 | - |  |  |  |  |
| 4. Withdrawal symptoms^a^ | .16* | -.50** | .70** | - |  |  |  |
| 5. Problematic pornography use | .73** | -.10 | .16* | .14 | - |  |  |
| 6. Past seven-day frequency of pornography use | .21** | -.06 | .00 | .02 | .20** | - |  |
| 7. Past four-week frequency of pornography use | .35** | -.05 | -.11 | .00 | .34** | .59** | - |

*Note*. ^a^Asked about the past seven days; *p<.05; **p<.01

**Table S6**

*Multilevel model results for manipulation check analyses*

| Outcome variable | Fixed effects | Estimate (*SE*) | *df* | Incidence rate ratios | Odds ratios | *t* | *z* | *p* | 95% CI |
| --- | --- | --- | --- | --- | --- | --- | --- | --- | --- |
| Abstinence effort^a^ | Group | -4.45 (0.48) | Inf | - | 0.01 | - | -9.37 | **<0.001** | 0.00 – 0.03 |
|  | Past seven-day abstinence effort | 0.19 (0.08) | Inf | - | 1.21 | - | 2.43 | **0.015** | 1.04 – 1.42 |
|  | Time | -0.04 (0.05) | Inf | - | 0.96 | - | -0.82 | 0.411 | 0.88 – 1.05 |
|  |  |  |  |  |  |  |  |  |  |
| FPU^b^ | Intercept | -2.21 (0.21) | Inf | 0.11 | - | - | -10.75 | **<0.001** | 0.07 – 0.16 |
|  | Group | 1.60 (0.17) | Inf | 4.95 | - | - | 9.38 | **<0.001** | 3.54 – 6.91 |
|  | Past seven-day FPU | 0.08 (0.02) | Inf | 1.09 | - | - | 4.21 | **<0.001** | 1.05 – 1.13 |
|  | Time | -0.05 (0.02) | Inf | 0.95 | - | - | -2.61 | **0.009** | 0.92 – 0.99 |
|  |  |  |  |  |  |  |  |  |  |
| Duration of pornography use | Intercept | 0.48 (1.77) | 245.03 | - | - | 0.27 | - | 0.787 | -2.97 – 3.93 |
|  | Group | 12.70 (1.82) | 171.76 | - | - | 6.97 | - | **<0.001** | 9.14 – 16.27 |
|  | Past seven-day duration of pornography use | 0.05 (0.01) | 176.24 | - | - | 5.35 | - | **<0.001** | 0.03 – 0.06 |
|  | Time | -0.46 (0.24) | 978.07 | - | - | -1.91 | - | 0.056 | -0.93 – 0.01 |
|  |  |  |  |  |  |  |  |  |  |

*Note*. ^a^Ordinal (fit using *clmm()* function). ^b^Poisson (fit using *glmer()* function). The random effect for the abstinence effort model was Time | Participant; the random effects for both FPU and duration of pornography use models were simplified to 1|Participant to facilitate model convergence.CI = confidence intervals; FPU = frequency of pornography use; Inf = infinity.

**Table S7**

*Means and standard deviations of all study variables for abstinence and control groups*

|  | Baseline | Day 1 | Day 2 | Day 3 | Day 4 | Day 5 | Day 6 | Day 7 |
| --- | --- | --- | --- | --- | --- | --- | --- | --- |
| Craving |  |  |  |  |  |  |  |  |
| Abstinence | 12.55 (3.40)^a^ | 7.05 (5.00) | 7.80 (6.07) | 8.26 (5.88) | 7.64 (6.13) | 6.30 (5.86) | 7.74 (6.49) | 8.00 (6.32) |
| Control | 11.51 (3.93)^a^ | 6.80 (4.29) | 7.11 (5.29) | 5.93 (4.27) | 6.23 (4.92) | 6.35 (5.17) | 6.04 (5.32) | 5.88 (5.10) |
| Total | 12.02 (3.70)^a^ | 6.92 (4.64) | 7.44 (5.67) | 7.08 (5.25) | 6.92 (5.58) | 6.33 (5.51) | 6.91 (5.99) | 6.92 (5.81) |
| Positive affect |  |  |  |  |  |  |  |  |
| Abstinence | 14.41 (3.47)^a^ | 13.99 (3.68) | 12.99 (4.04) | 13.29 (4.17) | 13.19 (4.22) | 13.04 (4.18) | 13.93 (4.37) | 14.05 (4.51) |
| Control | 14.39 (4.00)^a^ | 12.99 (4.35) | 13.03 (4.62) | 12.87 (4.34) | 13.03 (4.03) | 13.25 (4.55) | 13.07 (4.33) | 13.51 (4.80) |
| Total | 14.40 (3.74)^a^ | 13.48 (4.05) | 13.01 (4.34) | 13.08 (4.25) | 13.11 (4.12) | 13.14 (4.36) | 13.51 (4.36) | 13.78 (4.65) |
| Negative affect |  |  |  |  |  |  |  |  |
| Abstinence | 12.50 (4.14)^a^ | 9.96 (4.45) | 9.94 (4.16) | 9.42 (4.03) | 9.49 (4.28) | 8.81 (3.74) | 8.91 (3.50) | 9.22 (3.67) |
| Control | 12.24 (4.40)^a^ | 9.57 (4.12) | 9.57 (4.53) | 9.70 (4.55) | 9.00 (4.32) | 10.09 (4.78) | 9.35 (4.47) | 8.99 (4.00) |
| Total | 12.37 (4.26)^a^ | 9.77 (4.28) | 9.75 (4.35) | 9.56 (4.29) | 9.24 (4.30) | 9.46 (4.33) | 9.12 (4.00) | 9.10 (3.84) |
| Withdrawal symptoms |  |  |  |  |  |  |  |  |
| Abstinence | 2.15 (0.66)^a^ | 1.83 (0.73) | 1.82 (0.71) | 1.73 (0.75) | 1.74 (0.82) | 1.62 (0.74) | 1.71 (0.72) | 1.70 (0.72) |
| Control | 2.13 (0.74)^a^ | 1.92 (0.84) | 1.81 (0.78) | 1.80 (0.87) | 1.70 (0.83) | 1.80 (0.86) | 1.73 (0.86) | 1.71 (0.79) |
| Total | 2.14 (0.70)^a^ | 1.88 (0.79) | 1.82 (0.75) | 1.76 (0.81) | 1.72 (0.82) | 1.71 (0.81) | 1.72 (0.79) | 1.70 (0.75) |
| Abstinence effort |  |  |  |  |  |  |  |  |
| Abstinence | 1.06 (2.48)^a^ | 4.92 (3.89) | 4.40 (3.56) | 4.71 (3.65) | 4.51 (3.92) | 4.51 (3.73) | 4.94 (3.71) | 5.06 (3.99) |
| Control | 1.19 (2.51)^a^ | 1.06 (2.43) | 1.11 (2.57) | 1.14 (2.53) | 0.82 (2.26) | 1.04 (2.54) | 1.01 (2.48) | 0.50 (1.83) |
| Total | 1.13 (2.49)^a^ | 2.97 (3.76) | 2.69 (3.48) | 2.90 (3.60) | 2.64 (3.68) | 2.75 (3.62) | 3.01 (3.72) | 2.74 (3.83) |
| Frequency of pornography use (FPU) |  |  |  |  |  |  |  |  |
| Abstinence | 5.93 (4.49)^b^ | 0.20 (0.49) | 0.45 (0.99) | 0.33 (1.05) | 0.17 (0.47) | 0.20 (0.56) | 0.31 (0.86) | 0.25 (0.93) |
| Control | 5.42 (2.96)^b^ | 1.14 (1.35) | 0.93 (0.96) | 0.93 (1.11) | 0.98 (0.94) | 0.83 (0.93) | 1.03 (1.05) | 0.69 (0.82) |
| Total | 5.67 (3.78)^b^ | 0.68 (1.12) | 0.70 (1.00) | 0.63 (1.12) | 0.57 (0.84) | 0.51 (0.82) | 0.66 (1.02) | 0.47 (0.90) |
| Duration of pornography use |  |  |  |  |  |  |  |  |
| Abstinence | 108.88 (100.71)^b^ | 2.74 (8.14) | 7.70 (26.00) | 4.31 (12.72) | 3.43 (11.49) | 3.52 (16.61) | 3.90 (10.17) | 4.20 (15.33) |
| Control | 110.60 (109.75)^b^ | 20.54 (28.34) | 15.81 (21.74) | 16.71 (21.03) | 19.28 (24.60) | 15.91 (21.51) | 17.88 (24.81) | 12.26 (17.76) |
| Total | 109.76 (105.13)^b^ | 11.80 (22.77) | 11.85 (24.18) | 10.47 (18.39) | 11.31 (20.69) | 9.52 (20.06) | 10.57 (19.86) | 8.25 (17.03) |
| Frequency of masturbation without pornography |  |  |  |  |  |  |  |  |
| Abstinence | 1.84 (0.96)^c^ | 0.15 (0.53) | 0.21 (0.85) | 0.20 (0.72) | 0.17 (0.66) | 0.20 (1.00) | 0.22 (0.84) | 0.16 (0.51) |
| Control | 2.09 (1.36)^c^ | 0.06 (0.24) | 0.20 (0.55) | 0.15 (0.42) | 0.13 (0.47) | 0.08 (0.35) | 0.06 (0.34) | 0.07 (0.30) |
| Total | 1.97 (1.19)^c^ | 0.10 (0.41) | 0.21 (0.71) | 0.18 (0.59) | 0.15 (0.57) | 0.14 (0.76) | 0.15 (0.65) | 0.12 (0.42) |
| Frequency of alternative sexual activity |  |  |  |  |  |  |  |  |
| Abstinence | NA | 0.04 (0.19) | 0.05 (0.27) | 0.06 (0.36) | 0.09 (0.36) | 0.19 (1.35) | 0.00 (0.00) | 0.01 (0.11) |
| Control | NA | 0.01 (0.11) | 0.02 (0.15) | 0.11 (0.46) | 0.06 (0.33) | 0.01 (0.11) | 0.04 (0.25) | 0.00 (0.00) |
| Total | NA | 0.02 (0.15) | 0.04 (0.22) | 0.08 (0.41) | 0.07 (0.34) | 0.11 (0.97) | 0.02 (0.18) | 0.01 (0.08) |

*Note*. ^a^Asked about the past seven days; ^b^Asked about total frequency or duration during the past seven days; ^c^Asked about average frequency over the past four weeks on a non-equivalent scale to the daily measure; NA = not assessed

**Table S8**

*Model fit statistics of multilevel models in confirmatory analyses*

| Outcome variable | Model | Fixed effects | Random effects | AIC | BIC | logLik | Deviance | df |
| --- | --- | --- | --- | --- | --- | --- | --- | --- |
| Craving | 1 | Group × PPU + baseline craving + past four-week FPU + frequency of masturbation without pornography + frequency of alternative sexual activity + time | Time \| Participant | 6776.2 | 6841.8 | -3375.1 | 6750.2 | 13 |
|  | **2** | **Group + PPU + baseline craving + past four-week FPU + frequency of masturbation without pornography + frequency of alternative sexual activity + time** | **Time \| Participant** | **6775.6** | **6836.1** | **-3375.8** | **6751.6** | **12** |
|  | 3 | Group + baseline craving + past four-week FPU + frequency of masturbation without pornography + frequency of alternative sexual activity + time | Time \| Participant | 6792.2 | 6847.7 | -3385.1 | 6770.2 | 11 |
| Positive affect | 1 | Group × PPU + baseline positive affect + past four-week FPU + frequency of masturbation without pornography + frequency of alternative sexual activity + time | Time \| Participant | 5918.5 | 5984.1 | -2946.3 | 5892.5 | 13 |
|  | 2 | Group + PPU + baseline positive affect + past four-week FPU + frequency of masturbation without pornography + frequency of alternative sexual activity + time | Time \| Participant | 5918.1 | 5978.6 | -2947.1 | 5894.1 | 12 |
|  | **3** | **Group + baseline positive affect + past four-week FPU + frequency of masturbation without pornography + frequency of alternative sexual activity + time** | **Time \| Participant** | **5916.1** | **5971.6** | **-2947.1** | **5894.1** | **11** |
| Negative affect^a^ | 1 | Group × PPU + baseline negative affect + past four-week FPU + frequency of masturbation without pornography + frequency of alternative sexual activity + time | Time \| Participant | 507.4 | 573.0 | -240.7 | 481.4 | 13 |
|  | 2 | Group + PPU + baseline negative affect + past four-week FPU + frequency of masturbation without pornography + frequency of alternative sexual activity + time | Time \| Participant | 506.0 | 566.6 | -241.0 | 482.0 | 12 |
|  | **3** | **Group + baseline negative affect + past four-week FPU + frequency of masturbation without pornography + frequency of alternative sexual activity + time** | **Time \| Participant** | **507.2** | **562.6** | **-242.6** | **485.2** | **11** |
| Withdrawal symptoms | 1 | Group × PPU + baseline withdrawal symptoms + past four-week FPU + frequency of masturbation without pornography + frequency of alternative sexual activity + time | Time \| Participant | 2049.3 | 2114.8 | -1011.6 | 2023.3 | 13 |
|  | **2** | **Group + PPU + baseline withdrawal symptoms + past four-week FPU + frequency of masturbation without pornography + frequency of alternative sexual activity + time** | **Time \| Participant** | **2048.8** | **2109.3** | **-1012.4** | **2024.8** | **12** |
|  | 3 | Group + baseline withdrawal symptoms + past four-week FPU + frequency of masturbation without pornography + frequency of alternative sexual activity + time | Time \| Participant | 2053.3 | 2108.8 | -1015.6 | 2031.3 | 11 |

*Note*. ^a^log-transformed. AIC = Akaike information criterion; BIC = Bayesian information criterion; PPU = problematic pornography use. FPU = frequency of pornography use.

**Table S9**

*Model comparisons of multilevel models in confirmatory analyses*

| Outcome variable | Model comparison | *χ^2^* | *df* | *p* |
| --- | --- | --- | --- | --- |
| Craving | Model 1 vs. Model 2 | 1.35 | 1 | 0.245 |
|  | Model 2 vs. Model 3 | 18.64 | 1 | **<0.001** |
| Positive affect | Model 1 vs. Model 2 | 1.57 | 1 | 0.210 |
|  | Model 2 vs. Model 3 | 0.01 | 1 | 0.940 |
| Negative affect | Model 1 vs. Model 2 | 0.59 | 1 | 0.443 |
|  | Model 2 vs. Model 3 | 3.12 | 1 | 0.077 |
| Withdrawal symptoms | Model 1 vs. Model 2 | 1.50 | 1 | 0.220 |
|  | Model 2 vs. Model 3 | 6.51 | 1 | **0.011** |

**Table S10**

*Baseline characteristics of the sample by gender*

| Characteristic | Males  (*n* = 61) | Females  (*n* = 113) | Total  (*N* = 174) | Group difference |
| --- | --- | --- | --- | --- |
| *Past four-week frequency of pornography use^a^*, *n* (%) |  |  |  | **χ*^2 (^*5) = 45.615, *p*<0.001** |
| 3 times a week on average | 7 (11.48) | 55 (48.67) | 62 (35.63) |  |
| 4 times a week on average | 13 (21.31) | 32 (28.32) | 45 (25.86) |  |
| 5 times a week on average | 7 (11.48) | 12 (10.62) | 19 (10.92) |  |
| 6 times a week on average | 12 (19.67) | 3 (2.65) | 15 (8.62) |  |
| Once a day on average | 13 (21.31) | 9 (7.96) | 22 (12.64) |  |
| More than once a day on average | 9 (14.75) | 2 (1.77) | 11 (6.32) |  |
| *Past four-week frequency of pornography use^b^* (range 1 – 6), *M (SD)* | 3.62 (1.64) | 1.98 (1.31) | 2.56 (1.63) | **F(1,172)=51.768, *p*<0.001** |
| *Past four-week average duration of pornography use per session^a^*, *n* (%) |  |  |  | χ*^2 (^*7) = 5.885, *p* = 0.553 |
| 5 minutes or less | 2 (3.28) | 11 (9.73) | 13 (7.47) |  |
| 6 to 10 minutes | 12 (19.67) | 31 (27.43) | 43 (24.71) |  |
| 11 to 20 minutes | 18 (29.51) | 30 (26.55) | 48 (27.59) |  |
| 21 to 30 minutes | 14 (22.95) | 20 (17.70) | 34 (19.54) |  |
| 31 to 45 minutes | 10 (16.39) | 11 (9.73) | 21 (12.07) |  |
| 46 to 60 minutes | 3 (4.92) | 5 (4.42) | 8 (4.60) |  |
| 1 to 1.5 hours | 2 (3.28) | 4 (3.54) | 6 (3.45) |  |
| 1.5 to 2 hours | 0 (0.00) | 1 (0.88) | 1 (0.57) |  |
| *Past four-week average duration of pornography use per session^b^* (range 1 – 8), *M (SD)* | 3.57 (1.37) | 3.22 (1.55) | 3.34 (1.49) | F(1,172)=2.226, *p*=0.138 |
| *Past four-week frequency of masturbation without pornography^a^*, *n* (%) |  |  |  | χ*^2 (^*5) = 5.107, *p* = 0.403 |
| Never | 28 (45.90) | 50 (44.25) | 78 (44.83) |  |
| 3 times or less in total | 19 (31.15) | 38 (33.63) | 57 (32.76) |  |
| Once a week on average | 7 (11.48) | 11 (9.73) | 18 (10.34) |  |
| 2-3 times a week on average | 2 (3.28) | 11 (9.73) | 13 (7.47) |  |
| 4-6 times a week on average | 3 (4.92) | 2 (1.77) | 5 (2.87) |  |
| 7 or more times a week on average | 2 (3.28) | 1 (0.88) | 3 (1.72) |  |
| *Past four-week frequency of masturbation without pornography^b^* (range 1 – 6), *M (SD)* | 2.00 (1.30) | 1.94 (1.11) | 1.96 (1.18) | F(1,172)=0.109, *p*=0.742 |
| *Past four-week percentage of pornography sessions accompanied by masturbation* (range 0-100), *M (SD)* | 85.05 (21.60) | 60.82 (35.79) | 69.32 (33.55) | ***F*(1,172)=23.327, *p*<0.001** |
| *Past seven-day total frequency of pornography use, M (SD)* | 6.80 (3.66) | 5.05 (3.74) | 5.67 (3.80) | ***F*(1,172)=9.389, *p* = 0.003** |
| *Past seven-day frequency of pornography use without masturbation, M (SD)* | 1.52 (3.72) | 1.67 (2.79) | 1.62 (3.14) | *F*(1,172)=0.088, *p* = 0.768 |
| *Past seven-day frequency of pornography use with masturbation, M (SD)* | 5.28 (2.87) | 3.38 (3.42) | 4.05 (3.36) | ***F*(1,172)=13.587, *p*< 0.001** |
| *Past seven-day duration of pornography use in minutes, M (SD)* | 138.13 (109.36) | 92.67 (97.71) | 108.61 (103.94) | ***F*(1,172)=7.880, *p* = 0.006** |
| *Problematic pornography use* (range 0-126), *M (SD*) | 60.02 (18.22) | 51.42 (17.91) | 54.43 (18.43) | ***F*(1,172)=9.027, *p* = 0.003** |
| *Moral disapproval of pornography* (range 1-7), *M (SD)* | 2.69 (1.57) | 2.78 (1.62) | 2.75 (1.60) | *F*(1,172)=0.126, *p* = 0.723 |
| *Intrinsic desire to quit or reduce pornography use, n* (%) |  |  |  | *χ^2 (^*2) = 5.615, *p* = 0.060 |
| Reduce | 34 (55.74) | 51 (45.13) | 85 (48.85) |  |
| Quit | 9 (14.75) | 9 (7.96) | 18 (10.34) |  |
| No desire to reduce or quit | 18 (29.51) | 53 (46.90) | 71 (40.80) |  |
| *Abstinence effort, M (SD)* | 0.79 (2.04) | 1.33 (2.71) | 1.14 (2.50) | *F*(1,172)=1.859, *p* = 0.175 |
| *Craving for pornography* (range 0-24), *M (SD)* | 13.00 (3.75) | 11.45 (3.58) | 11.99 (3.71) | ***F*(1,172)=7.168, *p* = 0.008** |
| *Negative affect* (range 0-25), *M (SD)* | 11.39 (4.11) | 12.76 (4.20) | 12.28 (4.21) | ***F*(1,172)=4.264, *p* = 0.040** |
| *Positive affect* (range 0 - 25), *M (SD)* | 14.64 (3.67) | 14.28 (3.79) | 14.41 (3.74) | *F*(1,172)=0.358, *p* = 0.551 |
| *Withdrawal symptoms* (range 0-4), *M (SD)* | 2.01 (0.63) | 2.18 (0.72) | 2.12 (0.69) | *F*(1,172)=2.421, *p* = 0.122 |

*Note*. ^a^Treated as categorical variable. ^b^Treated as continuous variable. *M* = mean, *SD* = standard deviation.

**Table S11**

*Model fit statistics of multilevel models in exploratory analyses with past four-week FPU as moderator*

| Outcome variable | Model | Fixed effects | Random effects | AIC | BIC | logLik | Deviance | df |
| --- | --- | --- | --- | --- | --- | --- | --- | --- |
| Craving | **1** | **Group × PPU × past four-week FPU + baseline craving + frequency of masturbation without pornography + frequency of alternative sexual activity + time + gender** | **Time \| Participant** | **6698.3** | **6783.9** | **-3332.2** | **6664.3** | **17** |
|  | 2 | Group × past four-week FPU + PPU + baseline craving + frequency of masturbation without pornography + frequency of alternative sexual activity + time + gender | Time \| Participant | 6703.4 | 6773.9 | -3337.7 | 6675.4 | 14 |
| Positive affect | 1 | Group × PPU × past four-week FPU + baseline positive affect + frequency of masturbation without pornography + frequency of alternative sexual activity + time + gender | Time \| Participant | 5841.8 | 5927.3 | -2903.9 | 5807.8 | 17 |
|  | 2 | Group × past four-week FPU + PPU + baseline positive affect + frequency of masturbation without pornography + frequency of alternative sexual activity + time + gender | Time \| Participant | 5842.2 | 5912.6 | -2907.1 | 5814.2 | 14 |
|  | 3 | Group + past four-week FPU + PPU + baseline positive affect + frequency of masturbation without pornography + frequency of alternative sexual activity + time + gender | Time \| Participant | 5841.0 | 5906.4 | -2907.5 | 5815.0 | 13 |
|  | **4** | **Group + past four-week FPU + baseline positive affect + frequency of masturbation without pornography + frequency of alternative sexual activity + time + gender** | **Time \| Participant** | **5839.0** | **5899.4** | **-2907.5** | **5815.0** | **12** |
| Negative affect^a^ | 1 | Group × PPU × past four-week FPU + baseline negative affect + frequency of masturbation without pornography + frequency of alternative sexual activity + time + gender | Time \| Participant | 490.9 | 576.4 | -228.4 | 456.9 | 17 |
|  | 2 | Group × past four-week FPU + PPU + baseline negative affect + frequency of masturbation without pornography + frequency of alternative sexual activity + time + gender | Time \| Participant | 486.11 | 556.6 | -229.1 | 458.1 | 14 |
|  | **3** | **Group + past four-week FPU + PPU + baseline negative affect + frequency of masturbation without pornography + frequency of alternative sexual activity + time + gender** | **Time \| Participant** | **484.2** | **549.6** | **-229.1** | **458.2** | **13** |
|  | 4 | Group + past four-week FPU + baseline negative affect + frequency of masturbation without pornography + frequency of alternative sexual activity + time + gender | Time \| Participant | 486.4 | 546.8 | -231.2 | 462.4 | 12 |
| Withdrawal symptoms | 1 | Group × PPU × past four-week FPU + baseline withdrawal symptoms + frequency of masturbation without pornography + frequency of alternative sexual activity + time + gender | Time \| Participant | 2030.6 | 2116.1 | -998.3 | 1996.6 | 17 |
|  | 2 | Group × past four-week FPU + PPU + baseline withdrawal symptoms + frequency of masturbation without pornography + frequency of alternative sexual activity + time + gender | Time \| Participant | 2026.9 | 2097.4 | -999.5 | 1998.9 | 14 |
|  | **3** | **Group + past four-week FPU + PPU + baseline withdrawal symptoms + frequency of masturbation without pornography + frequency of alternative sexual activity + time + gender** | **Time \| Participant** | **2025.7** | **2091.1** | **-999.8** | **1999.7** | **13** |
|  | 4 | Group + past four-week FPU + baseline withdrawal symptoms + frequency of masturbation without pornography + frequency of alternative sexual activity + time + gender | Time \| Participant | 2031.1 | 2091.3 | -1003.5 | 2007.0 | 12 |

*Note*. ^a^log-transformed. AIC = Akaike information criterion; BIC = Bayesian information criterion; PPU = problematic pornography use. FPU = frequency of pornography use. Nelder Mead optimizer used for craving and withdrawal symptoms models due to non-convergence issues with default optimizer.

**Table S12**

*Model comparisons of multilevel models in exploratory analyses with past four-week FPU as moderator*

| Outcome variable | Model comparison | *χ^2^* | *df* | *p* |
| --- | --- | --- | --- | --- |
| Craving | Model 1 vs. Model 2 | 11.069 | 3 | **0.011** |
| Positive affect | Model 1 vs. Model 2 | 6.431 | 3 | 0.092 |
|  | Model 2 vs. Model 3 | 0.798 | 1 | 0.372 |
|  | Model 3 vs. Model 4 | 0.062 | 1 | 0.803 |
| Negative affect | Model 1 vs. Model 2 | 1.260 | 3 | 0.739 |
|  | Model 2 vs. Model 3 | 0.062 | 1 | 0.804 |
|  | Model 3 vs. Model 4 | 4.228 | 1 | **0.040** |
| Withdrawal symptoms | Model 1 vs. Model 2 | 2.351 | 3 | 0.503 |
|  | Model 2 vs. Model 3 | 0.738 | 1 | 0.390 |
|  | Model 3 vs. Model 4 | 7.297 | 1 | **0.007** |

**Table S13**

*Contrasts between abstinence and control groups at combinations of high (+1 SD) or low (-1 SD) PPU and all six levels of past four-week FPU*

| Outcome variable | PPU level | Past four-week FPU | Group | Estimated marginal mean (*SE*) | Estimate (*SE*)  (Contrast:  Abstinence vs Control) | *df* | *t* | *p*^a^ |
| --- | --- | --- | --- | --- | --- | --- | --- | --- |
| Craving | Low | 3 times a week | Abstinence | 5.31 (0.72) | 0.82 (0.85) | 160 | 0.96 | 0.453 |
|  |  |  | Control | 4.49 (0.69) |  |  |  |  |
|  | Low | 4 times a week | Abstinence | 5.48 (0.57) | 0.95 (0.68) | 161 | 1.39 | 0.334 |
|  |  |  | Control | 4.53 (0.57) |  |  |  |  |
|  | Low | 5 times a week | Abstinence | 5.65 (0.67) | 1.08 (0.83) | 163 | 1.30 | 0.334 |
|  |  |  | Control | 4.57 (0.60) |  |  |  |  |
|  | Low | 6 times a week | Abstinence | 5.82 (0.95) | 1.21 (1.18) | 163 | 1.03 | 0.453 |
|  |  |  | Control | 4.61 (0.77) |  |  |  |  |
|  | Low | Once a day | Abstinence | 5.98 (1.29) | 1.34 (1.60) | 163 | 0.84 | 0.484 |
|  |  |  | Control | 4.64 (1.00) |  |  |  |  |
|  | Low | More than once a day | Abstinence | 6.15 (1.66) | 1.47 (2.06) | 163 | 0.72 | 0.518 |
|  |  |  | Control | 4.68 (1.26) |  |  |  |  |
|  | High | 3 times a week | Abstinence | 6.19 (0.77) | -3.38 (1.11) | 163 | -3.04 | **0.016** |
|  |  |  | Control | 9.57 (0.90) |  |  |  |  |
|  | High | 4 times a week | Abstinence | 7.48 (0.57) | -1.87 (0.84) | 162 | -2.23 | 0.082 |
|  |  |  | Control | 9.35 (0.72) |  |  |  |  |
|  | High | 5 times a week | Abstinence | 8.77 (0.50) | -0.36 (0.70) | 161 | -0.52 | 0.604 |
|  |  |  | Control | 9.13 (0.62) |  |  |  |  |
|  | High | 6 times a week | Abstinence | 10.05 (0.59) | 1.14 (0.77) | 160 | 1.49 | 0.333 |
|  |  |  | Control | 8.91 (0.64) |  |  |  |  |
|  | High | Once a day | Abstinence | 11.34 (0.79) | 2.65 (1.00) | 160 | 2.65 | **0.036** |
|  |  |  | Control | 8.69 (0.77) |  |  |  |  |
|  | High | More than once a day | Abstinence | 12.62 (1.04) | 4.16 (1.31) | 161 | 3.17 | **0.016** |
|  |  |  | Control | 8.47 (0.97) |  |  |  |  |

*Note*. ^a^False discovery rate (FDR) adjusted.

**Table S14**

*Multilevel model results for all outcome variables in exploratory analyses with time as moderator*

| Outcome variable | Fixed effect | Estimate (*SE*) | *df* | *t* | *p* | 95% CI |
| --- | --- | --- | --- | --- | --- | --- |
| Craving | Intercept | -0.14 (1.19) | 163.30 | -0.12 | 0.908 | -2.48 – 2.20 |
|  | Group | -0.35 (0.50) | 163.37 | -0.70 | 0.486 | -1.33 – 0.63 |
|  | Time (linear) | -3.61 (5.12) | 169.11 | -0.71 | 0.482 | -13.65 – 6.43 |
|  | Time (quadratic) | 0.20 (4.23) | 817.47 | 0.05 | 0.963 | -8.09 – 8.49 |
|  | PPU | 0.09 (0.02) | 161.73 | 4.78 | **<0.001** | 0.06 – 0.13 |
|  | Past four-week FPU | 0.32 (0.18) | 166.99 | 1.79 | 0.076 | -0.03 – 0.68 |
|  | Gender | 0.26 (0.59) | 163.92 | 0.45 | 0.656 | -0.89 – 1.41 |
|  | Baseline craving | 0.07 (0.10) | 161.14 | 0.69 | 0.493 | -0.12 – 0.26 |
|  | Frequency of masturbation without pornography | 0.44 (0.30) | 924.51 | 1.47 | 0.142 | -0.15 – 1.03 |
|  | Frequency of alternative sexual activity | -0.33(0.34) | 996.40 | -0.99 | 0.324 | -1.00 – 0.33 |
| Positive affect | Intercept | 4.70 (1.33) | 169.54 | 3.54 | **<0.001** | 2.09 – 7.30 |
|  | Group | -0.38 (0.41) | 165.86 | -0.91 | 0.363 | -1.19 – 0.43 |
|  | Time (linear) | 4.76 (3.62) | 167.48 | 1.31 | 0.191 | -2.35 – 11.86 |
|  | Time (quadratic) | 8.94 (2.73) | 818.61 | 3.28 | **0.001** | 3.59 – 14.29 |
|  | PPU | 0.00 (0.01) | 165.96 | 0.23 | 0.818 | -0.02 – 0.03 |
|  | Past four-week FPU | 0.00 (0.15) | 168.12 | 0.02 | 0.982 | -0.29 – 0.30 |
|  | Gender | 0.14 (0.49) | 166.22 | 0.29 | 0.774 | -0.82 – 1.10 |
|  | Baseline positive affect | 0.60 (0.06) | 169.32 | 10.76 | **<0.001** | 0.49 – 0.70 |
|  | Frequency of masturbation without pornography | -0.22 (0.21) | 1030.81 | -1.07 | 0.283 | -0.62 – 0.18 |
|  | Frequency of alternative sexual activity | 0.23 (0.23) | 965.09 | 1.04 | 0.298 | -0.21 – 0.67 |
| Negative affect^a^ | Intercept | 1.25 (0.10) | 160.00 | 12.20 | **<0.001** | 1.05 – 1.46 |
|  | Group | 0.01 (0.04) | 160.87 | 0.34 | 0.738 | -0.06 – 0.09 |
|  | Time (linear) | -0.89 (0.34) | 167.30 | -2.60 | **0.010** | -1.56 – -0.22 |
|  | Time (quadratic) | 0.18 (0.26) | 817.82 | 0.68 | 0.495 | -0.33 – 0.68 |
|  | PPU | 0.00 (0.00) | 161.38 | 2.09 | **0.039** | 0.00 – 0.00 |
|  | Past four-week FPU | 0.01 (0.01) | 162.45 | 1.00 | 0.318 | -0.01 – 0.04 |
|  | Gender | 0.10 (0.05) | 161.43 | 2.19 | **0.030** | 0.01 – 0.20 |
|  | Baseline negative affect | 0.05 (0.00) | 161.99 | 10.76 | **<0.001** | 0.04 – 0.06 |
|  | Frequency of masturbation without pornography | -0.00 (0.02) | 1045.79 | -0.07 | 0.945 | -0.04 – 0.04 |
|  | Frequency of alternative sexual activity | 0.02 (0.02) | 962.22 | -0.89 | 0.372 | -0.02 – 0.06 |
| Withdrawal symptoms | Intercept | 0.10 (0.18) | 160.75 | 0.55 | 0.585 | -0.26 – 0.46 |
|  | Group | 0.10 (0.07) | 161.48 | 1.34 | 0.183 | -0.04 – 0.24 |
|  | Time (linear) | -2.10 (0.66) | 166.55 | -3.16 | **0.002** | -3.41 – -0.80 |
|  | Time (quadratic) | 0.84 (0.52) | 819.77 | 1.62 | 0.106 | -0.18 – 1.85 |
|  | PPU | 0.01 (0.00) | 162.22 | 2.75 | **0.007** | 0.00 – 0.01 |
|  | Past four-week FPU | -0.00 (0.03) | 163.66 | -0.15 | 0.882 | -0.05 – 0.05 |
|  | Gender | 0.15 (0.09) | 161.93 | 1.76 | 0.081 | -0.02 – 0.32 |
|  | Baseline withdrawal symptoms | 0.57 (0.05) | 166.33 | 10.82 | **<0.001** | 0.46 – 0.67 |
|  | Frequency of masturbation without pornography | 0.03 (0.04) | 1013.71 | 0.69 | 0.493 | -0.05 – 0.10 |
|  | Frequency of alternative sexual activity | -0.04 (0.04) | 977.92 | -0.85 | 0.396 | -0.12 – 0.05 |

*Note*. ^a^log-transformed. One participant in the abstinence group and one participant in the control group who identified as ‘agender’ were excluded from these analyses, resulting in *N* = 174. All models are random slope models with Time|Participant random effects. The best fitting models are presented. CI = confidence interval; FPU = frequency of pornography use; PPU = problematic pornography use; SE = standard error

**Table S15**

*Model fit statistics of multilevel models in exploratory analyses with time as moderator*

| Outcome variable | Model | Fixed effects | Random effects | AIC | BIC | logLik | Deviance | df |
| --- | --- | --- | --- | --- | --- | --- | --- | --- |
| Craving | 1 | (Group × PPU × time [quadratic]) + (group × PPU × time [linear]) + past four-week FPU + baseline craving + frequency of masturbation without pornography + frequency of alternative sexual activity + gender | Time \| Participant | 6711.2 | 6816.9 | -3334.6 | 6669.2 | 21 |
|  | 2 | (Group × time [quadratic]) + (group × time [linear]) + PPU + past four-week FPU + baseline craving + frequency of masturbation without pornography + frequency of alternative sexual activity + gender | Time \| Participant | 6712.2 | 6792.7 | -3340.1 | 6680.2 | 16 |
|  | **3** | **Group + time (quadratic) + time (linear) + PPU + past four-week FPU + baseline craving + frequency of masturbation without pornography + frequency of alternative sexual activity + gender** | **Time \| Participant** | **6710.4** | **6780.8** | **-3341.2** | **6682.4** | **14** |
| Positive affect | 1 | (Group × PPU × time [quadratic]) + (group × PPU × time [linear]) + past four-week FPU + baseline positive affect + frequency of masturbation without pornography + frequency of alternative sexual activity + gender | Time \| Participant | 5835.2 | 5940.9 | -2896.6 | 5793.2 | 21 |
|  | 2 | (Group × time [quadratic]) + (group × time [linear]) + PPU + past four-week FPU + baseline positive affect + frequency of masturbation without pornography + frequency of alternative sexual activity + gender | Time \| Participant | 5834.5 | 5915.0 | -2901.2 | 5802.5 | 16 |
|  | **3** | **Group + time (quadratic) + time (linear) + PPU + past four-week FPU + baseline positive affect + frequency of masturbation without pornography + frequency of alternative sexual activity + gender** | **Time \| Participant** | **5832.3** | **5902.8** | **-2902.2** | **5804.3** | **14** |
| Negative affect^a^ | 1 | (Group × PPU × time [quadratic]) + (group × PPU × time [linear]) + past four-week FPU + baseline negative affect + frequency of masturbation without pornography + frequency of alternative sexual activity + gender | Time \| Participant | 487.6 | 593.3 | -222.8 | 445.6 | 21 |
|  | 2 | (Group × time [quadratic]) + (group × time [linear]) + PPU + past four-week FPU + baseline negative affect + frequency of masturbation without pornography + frequency of alternative sexual activity + gender | Time \| Participant | 488.49 | 569.01 | -228.2 | 456.49 | 16 |
|  | **3** | **Group + time (quadratic) + time (linear) + PPU + past four-week FPU + baseline negative affect + frequency of masturbation without pornography + frequency of alternative sexual activity + gender** | **Time \| Participant** | **485.7** | **556.2** | **-228.9** | **457.7** | **14** |
| Withdrawal symptoms | 1 | (Group × PPU × time [quadratic]) + (group × PPU × time [linear]) + past four-week FPU + baseline withdrawal symptoms + frequency of masturbation without pornography + frequency of alternative sexual activity + gender | Time \| Participant | 2029.2 | 2134.9 | -993.6 | 1987.2 | 21 |
|  | 2 | (Group × time [quadratic]) + (group × time [linear]) + PPU + past four-week FPU + baseline withdrawal symptoms + frequency of masturbation without pornography + frequency of alternative sexual activity + gender | Time \| Participant | 2028.8 | 2109.3 | -998.4 | 1996.8 | 16 |
|  | **3** | **Group + time (quadratic) + time (linear) + PPU + past four-week FPU + baseline withdrawal symptoms + frequency of masturbation without pornography + frequency of alternative sexual activity + gender** | **Time \| Participant** | **2025.0** | **2095.5** | **-998.5** | **1997.0** | **14** |

*Note*. ^a^log-transformed. AIC = Akaike information criterion; BIC = Bayesian information criterion; PPU = problematic pornography use. FPU = frequency of pornography use. Nelder Mead optimizer used for negative affect model due to non-convergence issues with default optimizer.

**Table S16**

*Model comparisons of multilevel models in exploratory analyses with time as moderator*

| Outcome variable | Model comparison | *χ^2^* | *df* | *p* |
| --- | --- | --- | --- | --- |
| Craving | Model 1 vs. Model 2 | 11.028 | 5 | 0.051 |
|  | Model 2 vs. Model 3 | 2.182 | 2 | 0.336 |
| Positive affect | Model 1 vs. Model 2 | 9.333 | 5 | 0.096 |
|  | Model 2 vs. Model 3 | 1.795 | 2 | 0.408 |
| Negative affect | Model 1 vs. Model 2 | 10.921 | 5 | 0.053 |
|  | Model 2 vs. Model 3 | 1.214 | 2 | 0.545 |
| Withdrawal symptoms | Model 1 vs. Model 2 | 9.560 | 5 | 0.089 |
|  | Model 2 vs. Model 3 | 0.286 | 2 | 0.867 |

**References used in this supplementary document**

Hu, L. T., & Bentler, P. M. (1999). Cutoff criteria for fit indexes in covariance structure

analysis: Conventional criteria versus new alternatives. *Structural Equation Modeling: A Multidisciplinary Journal*, *6*(1), 1-55. <https://doi.org/10.1080/10705519909540118>

Kenny, D. A., Kaniskan, B., & McCoach, D. B. (2015). The performance of RMSEA in

models with small degrees of freedom. *Sociological Methods and Research*, *44*(3), 486–507. <https://doi.org/10.1177/0049124114543236>

Mueller, R. O., & Hancock, G. R. (2010). Structural equation modeling. In G. R. Hancock &

R. O. Mueller (Eds.), *The* *reviewer’s guide to quantitative methods in the social sciences* (pp. 371–384). New York, NY: Routledge.

Rosseel, Y. (2012). lavaan: An R Package for Structural Equation Modeling. *Journal of*

*Statistical Software, 48*(2), 1-36. <https://doi.org/10.18637/jss.v048.i02>
